# Crosstalk of *PmCBFs* and *PmDAMs* Based on the Changes of Phytohormones under Seasonal Cold Stress in the Stem of *Prunus mume*

**DOI:** 10.3390/ijms19020015

**Published:** 2018-01-23

**Authors:** Kai Zhao, Yuzhen Zhou, Yushu Li, Xiaokang Zhuo, Sagheer Ahmad, Yu Han, Xue Yong, Qixiang Zhang

**Affiliations:** 1 Beijing Key Laboratory of Ornamental Plants Germplasm Innovation & Molecular Breeding, National Engineering Research Center for Floriculture, Beijing Laboratory of Urban and Rural Ecological Environment, Key Laboratory of Genetics and Breeding in Forest Trees and Ornamental Plants of Ministry of Education, School of Landscape Architecture, Beijing Forestry University, 100083 Beijing, China; zhaokai87.sleep@gmail.com (K.Z.); zhouyuzhencn@163.com (Y.Z.); flyangel_girl@163.com (Y.L.); fjzhuoxk@126.com (X.Z.); sagheerhortii@gmail.com (S.A.); hanyu19880514@126.com (Y.H.); yongxue@bjfu.edu.cn (X.Y.); 2 Beijing Advanced Innovation Center for Tree Breeding by Molecular Design, Beijing Forestry University, 100083 Beijing, China

**Keywords:** *CBF* genes, *DAM* genes, *Prunus mume*, stem growth, expression analysis, Phytohormone assays

## Abstract

Plants facing the seasonal variations always need a growth restraining mechanism when temperatures turn down. C-repeat binding factor (*CBF*) genes work essentially in the cold perception. Despite lots of researches on *CBFs*, the multiple crosstalk is still interesting on their interaction with hormones and dormancy-associated MADS (DAM) genes in the growth and dormancy control. Therefore, this study highlights roles of *PmCBFs* in cold-induced dormancy from different orgens. And a sense-response relationship between *PmCBFs* and *PmDAMs* is exhibited in this process, jointly regulated by six *PmCBFs* and *PmDAM4–6*. Meantime, GA3 and ABA showed negative and positive correlation with *PmCBFs* expression levels, respectively. We also find a high correlation between IAA and *PmDAM1–3.* Finally, we display the interaction mode of PmCBFs and PmDAMs, especially PmCBF1-PmDAM1. These results can disclose another view of molecular mechanism in plant growth between cold-response pathway and dormancy regulation together with genes and hormones.

## 1. Introduction

*Prunus mume* was domesticated in northern China due to its cold resistance and adaptability to severe conditions. Bud dormancy is a crucial capacity of perennial plants that ensures survival during hard seasons. Recent genomic and transcriptomic investigations have brought to light fundamental mechanisms of molecular dormancy regulations [1,2]; however, the involvement of multiple hormones remains pluralistic, particularly in the fruit-bearing plants [3]. 

For woody plants, stem growth is significantly affected by low temperatures. Plants coordinate lifecycles in accordance with the seasonal shifts. Thus, they are affected by changes in temperatures. Central to this process is the ability to integrate environmental information into the vegetative growth rhythm. Understanding how plants regulate the timing of stem development has been given much attention, especially among deciduous trees, such as *Prunus*. Evidences indicate that some aspects of plant development are regulated by the MADS-box genes (Minichromosome maintenance 1 in yeast, AGAMOUS in *Arabidopsis*, DEFICIENS in snapdragon and serum response factor from human) and APETALA2/ethylene response factor (AP2/ERF) gene families, which integrate hormonal signals such as auxin, gibberellin, cytokinin and abscisic acid [4,5,6,7]. According to recent studies, *ERF* genes increase cell division and are important for ethylene signalling in vascular tissues. *ERF* family members also play vital roles in many other processes, including growth and development, dormancy and dormancy release, and biotic and abiotic stress responses [8,9,10]. Thus, combining analysis of genes expression and hormone levels may provide deeper understanding of the process of stem development. Additionally, acclimation enables plants to endure cold stress and is controlled by a mass of genes. *AtCBFs* (1–3) are key transcription factors directing the expression of specific groups of genes [11]. In cold climates, *CBF* genes play a prominent role in cold-response pathways, while *DAM* genes are related to plant dormancy and dormancy releasing. Because of their significant effects on plant development control, understanding their functions in stem development in *Prunus* can provide further value. 

*CBF* genes, also named *DREB* genes, belong to AP2/ERF gene family [12,13]. *CBF/DREB1* genes have been studied in various plant species, such as *Arabidopsis thaliana*, *Oryza sativa*, soybean (*Glycine max*), *Chrysanthemum*, *Vitis vinifera*, *Malus domestica*, *Prunus persica* and *Prunus mume* [14,15,16,17,18,19]. There are six *CBF/DREB1* genes in the model plant *Arabidopsis*, *CBF3/DREB1A* regulates many genes related to cold response, and hormone metabolism [20]. *DREB1* (*AtCBF1–3*) and *DREB2* also function in the signal pathway under low-temperature conditions [21]. The transgenic plants overexpressing *AtCBF1* and *3* show strong tolerance to cold stresses [22,23]. Unlike *AtCBF1–3*, the expression of *AtCBF4* is induced by abscisic acid (ABA) and is not influenced by low temperatures [24]. What’s more, *DDF1* and *DDF2* (*AtCBF5* and *AtCBF6*) are induced by salinity stress, which is connected to gibberellic acid (GA) levels and results in dwarfism and delayed flowering [25,26]. Cold hardiness has also been observed when a *PpCBF* from peach (*Prunus persica*) is overexpressed in apple [27]. In peach, there are five *CBF* genes, which are highly induced by 4 °C in leaves and this depends on specific *CBF* gene [27,28,29,30]. For other species, such as *Triticum aestivum* and *O. sativa*, the dwarf phenotype is observed in transgenic plants [14,31]. 

*DAM* genes act downstream for CBF genes and play a positive role in plant dormancy induction [8,32,33]. In *P. mume*, there are six *DAM* genes [34,35,36]. *PmDAMs* participate in endodormancy-associated expression [37,38]. The transcript accumulations of *DAM5* and *DAM6* in peach reach peaks, coinciding with the winter solstice and may be regulated by cold exposure [39]. In Japanese pear (*Pyrus pyrifolia*), *PpCBF2* regulates the expression of *PpMADS13–1*, which is a *DAM* gene [40]. Therefore, analyzing the relationship between *CBF* and *DAM* genes could aid research for stem growth in *Prunus*. 

In this study, six *PmCBFs* were cloned and the phylogenetic relationships between them were investigated. Then expression profiles for these genes in seven types of tissues were generated. To assess the interactive roles of *PmCBFs* and *PmDAMs*, and phytohormones in different processes of stem growth, the gene expression levels and varying hormonal concentrations were examined in stems. Finally, we reported the interaction modes between PmCBFs and PmDAMs in the dormancy of stem. The results of this study will contribute to a clearer understand of cold-response pathway and dormancy in plant. 

## 2. Results

### 2.1. Identification and Cloning of CBF Genes in P. mume

**Figure 1:**
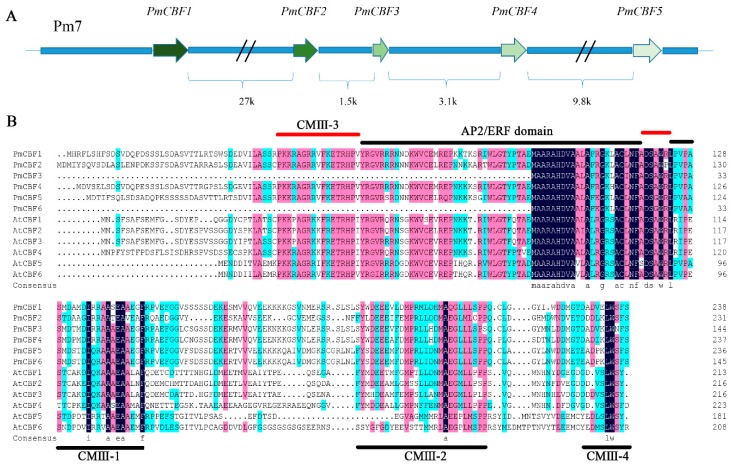
*CBF* genes in *P. mume*. (**A**) Gene locations of *PmCBFs* on chromosome 7; (**B**) Multiple sequences alignment of CBF proteins from *P. mume* and other species. Multiple sequence alignment of the CBF genes was applied using DNAMAN 7.0 program (Lynnon Corp., Quebec, QC, Canada). The AP2/ERF domain and CMIII domain (CMIII-1, CMIII-2, and CMIII-4) were shown by lines on bottom of the alignment. CMIII-3 was indicated by the red lines. The protein sequences of these CBF genes were shown in Supplementary Data S3.

Six *PmCBFs* were identified from the *Prunus mume* protein database (Supplementary Table S1). And *PmCBFs1–5* were found as a tandem array on Chromosome 7 (Figure 1A). The lengths of the sequences for *PmCBF1–6* were 720 bp, 699 bp, 438 bp, 717 bp, 714 bp, and 441 bp, and encoded 239, 232, 145, 238, 237, and 146 amino acids, respectively (Supplementary Data S1). These *PmCBFs* were highly consistent among their homologous genes with conserved AP2/ERF domains (Supplementary Figure S1). Then, sequences and conserved domains were verified by the InterPro website. 

### 2.2. Multiple Sequence Alignments and Phylogenetic Analysis

To carry out multiple sequence alignments, the protein sequences of six PmCBFs and 29 CBF genes from other plants were analysed. The genomic arrangement and related sequence structures were displayed in Figure 1. Especially the conserved domains, together with six AtCBFs, were marked (Figure 1B). At the N-terminals of the PmCBFs, the AP2/ERF domain exhibited high conservation, but the AP2/ERF domains of PmCBF3 and PmCBF6 were incomplete. In PmCBFs, CMIII domains, containing CMIII-1, CMIII-2, CMIII-3, and CMIII-4 motifs, were conserved. As shown in Figure 1B, CMIII-1 was behind the AP2/ERF domain; CMIII-3 had two regions on the both sides of the AP2/ERF domain; and CMIII-2 and CMIII-4 were at the C-terminals of protein sequences.

**Figure 2:**
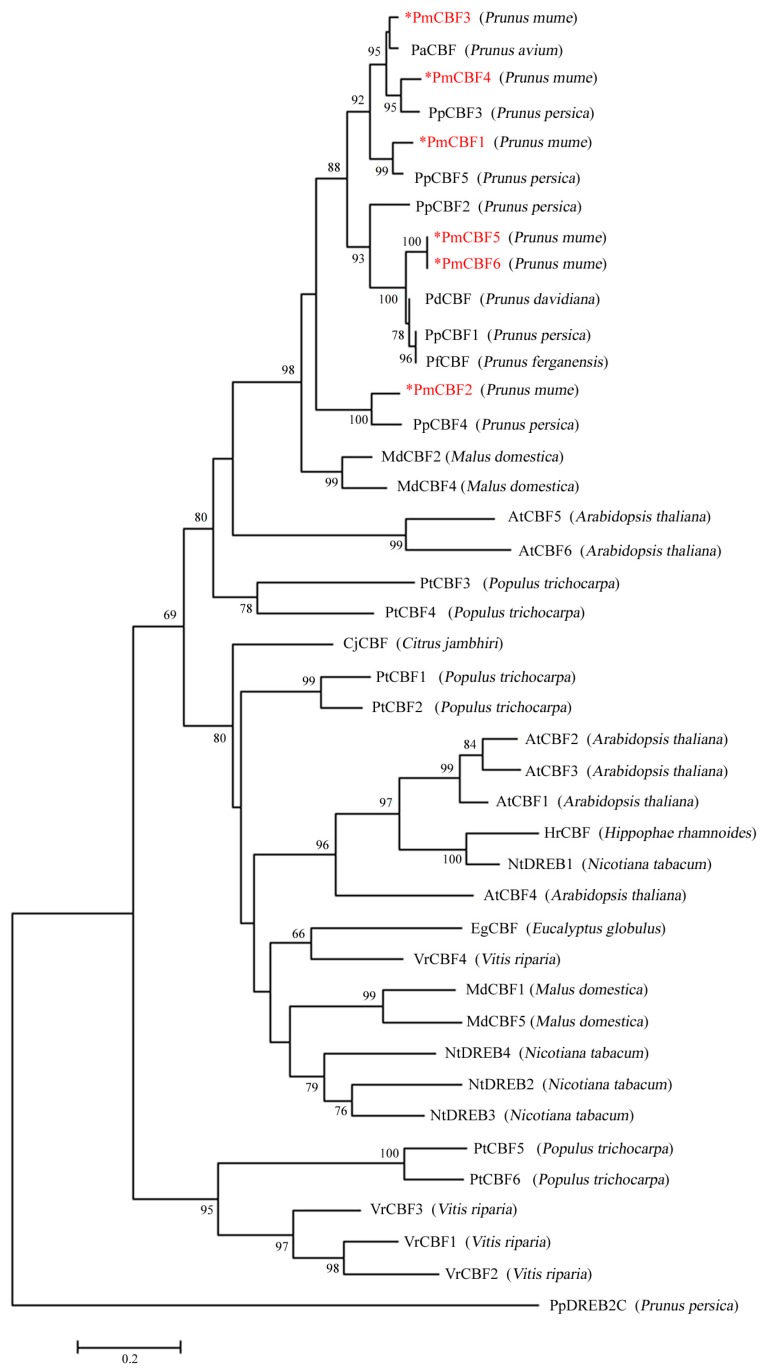
Phylogenetic tree of CBF proteins from *P. mume* and other species. This tree was constructed using MEGA7.1 with the Maximum-likelihood (ML) method. The sequences of these proteins were provided in Supplementary Data S3. Numbers above branches represented bootstrap values and the bootstrap values which below 60 were not shown. Stars (*) presented the *CBF* genes in *Prunus mume.*

To elucidate the relationship among CBF proteins, a phylogenetic tree was generated (Figure 2). In this dendrogram, six PmCBFs were first gathered with CBF genes from other *Prunus* plants (i.e., *P. persica*, *P. ferganensis*, *P. davidiana*, and *P. avium*), and then clustered with genes from other species (Figure 2). Six PmCBFs were converged with other *Prunus* CBF genes and formed three different evolutionary branches. *PmCBF5* and *PmCBF6* were gathered in one branch, while *PmCBF1*, *PmCBF3* and *PmCBF4* were in another branch. All of these genes were clustered with *PmCBF2*. Main CBFs in *Prunus* gathered in one branch from the branch of AtCBF5–6. But other CBFs from Nicotiana, *Populus tricocapa, Vitis riparia* obtained a closer relationship with AtCBF1–4.

### 2.3. Expressions of PmCBFs in Different Organs

**Figure 3:**
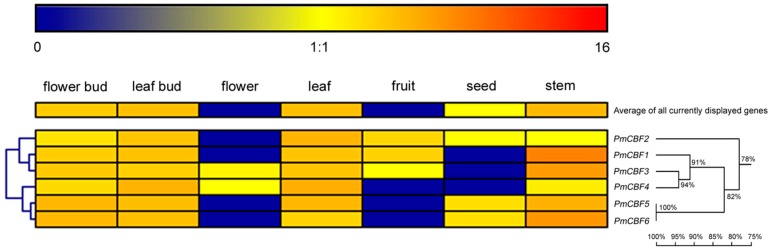
Expression profiles of *CBF* genes in different organs of *P. mume*. The expression levels of *PmCBFs* were obtained using RT-qPCR method. The colour scale represented the log2-transformed counts of the expression levels of *PmCBFs*. *Blue* indicated low expression levels and *Red* suggested high levels. Six *PmCBFs* were used to build the homology tree. Numbers above branches represented bootstrap value.

To measure the roles of *PmCBFs* in different organs (flower bud, leaf bud, flower, leaf, fruit, seed, and stem), the expression patterns were observed using the real-time quantitative PCR (RT-qPCR) and displayed by a heat map (Figure 3). Six *PmCBFs* were expressed in seven organs. These genes were highly detected in stem, moderately expressed in flower bud, leaf bud, and leaf, and poorly expressed in flower, fruit, and seed. According to the hierarchical clustering performed by Genesis software, *PmCBFs* were divided into two groups. *PmCBF5* and *PmCBF6* were gathered in one group. These two genes were predominantly detected in flower bud, leaf bud, leaf, and stem; and were slightly expressed in flower, fruit, and seed. The other group contained *PmCBF1*, *PmCBF2*, *PmCBF3*, and *PmCBF4*. The expression levels of these four genes were notable in flower bud, leaf bud, leaf, and stem. However, there were bare expressions for *PmCBF4–6* in fruit, *PmCBF1–2* and *5–6* in flower, *PmCBF1 and 3–4* in seed.

### 2.4. Sectionalization of PmCBFs and PmDAMs during the Expression in the Stem

**Figure 4:**
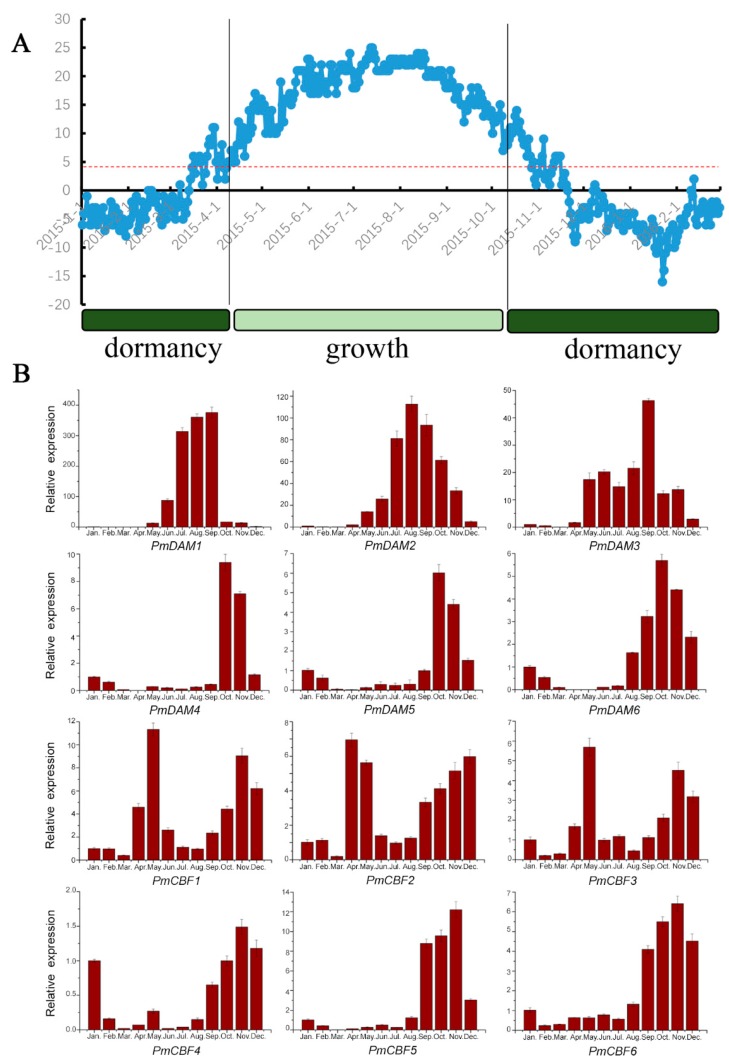
(**A**) The lowest day temperatures and growth statuses of *Prunus mume* stems; (**B**) Corresponding expression patterns of *PmCBFs* and *PmDAMs* during different periods of stem development. All RT-qPCR experiments were employed with three biological duplications, and each duplication was repeated in triplicate. The standard deviation of the results was shown by the error bars.

In Beijing (40°00′ N, 116°18′ E), the vegetation process occurs from April to September. The stem of *P. mume* ‘Sanlun Yudie’ grows rapidly in April, May, and June, becomes dormant in October and November, enters endo-dormancy from December to January and experiences dormancy release in February and March (Figure 4A). And six *PmCBFs* and six *PmDAMs* exhibited different expression levels in response to the cold stress from September to October when the stem fell into dormancy (Figure 4B). 

During the annual stem-growth process, the expression patterns of *PmCBF* genes showed significant specificity in the low-temperature climates (Figure 4B). The transcripts of *PmCBFs* manifested two similar expression trends and formed two groups. One group (*PmCBF1*, *PmCBF2*, and *PmCBF3*) was notably detected twice in the whole year: one expression peak occurred in May and the other in November, the appearance of expression peaks may be affected by the short coldness in the early spring and continuous low-temperature climates in winter. The other group, *PmCBF4–6,* established one expression peak together with the first group. All the expression of *PmCBFs* remarkably increased from August to November and decreased from December to March, indicating an active character in cold-response and dormancy control. *PmCBF1–3* acted more sensitively to the stress of cold than *PmCBF4–6*.

With high inner correlations of *PmDAM1–3* and *PmDAM4–6*, respectively, *PmDAMs* were divided into two groups based on different expression profiles (Figure 4B). The group consisting of *PmDAM1–3* had significant expression levels in August and September. The expressions of these three genes were intense in May to November and had low levels from January to April. The transcripts of these genes were up-regulated from May to September, but down-regulated in September to December. *PmDAM4–6* formed the second group, which showed strong expressions from September to December, with the highest expression level occurring in October. These genes also exhibited a rising expression trend from August to October, and gradually declined from October to February. The same genes were minimally expressed from March to July. These results demonstrated that these three genes might have positive correlations during dormancy induction and play a negative role in dormancy releasing.

### 2.5. Seasonal Changes in Hormones Synthesis and Correlations with Gene Expression during Stem Development

**Figure 5:**
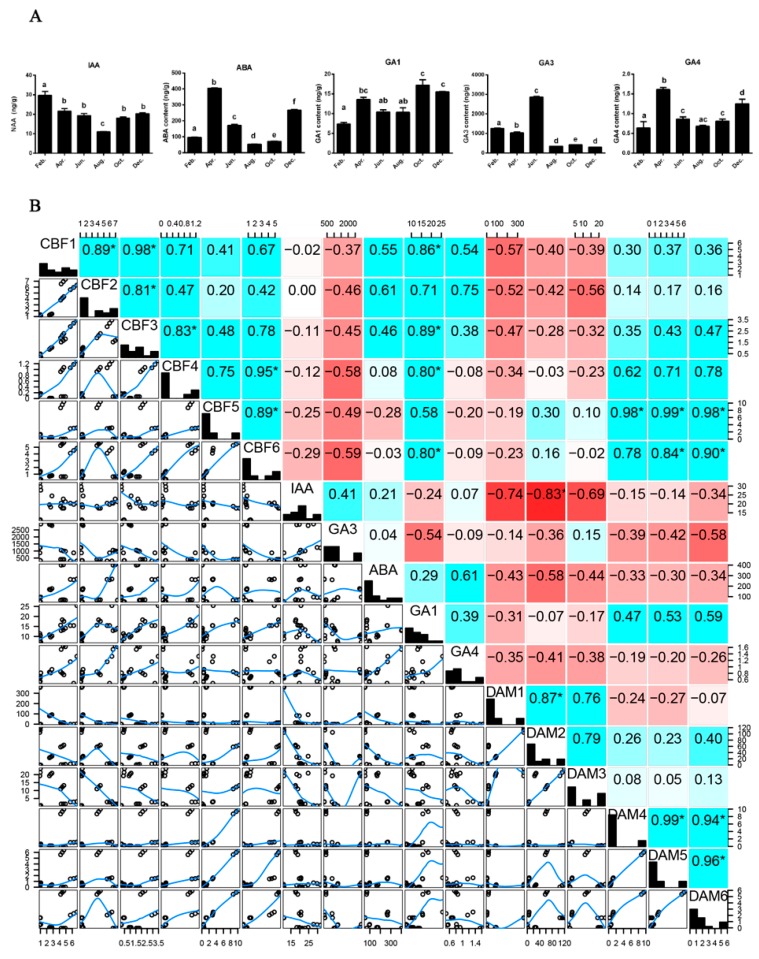
Hormone contents in stems and correlation matrix between hormones and genes. (**A**) the levels of IAA, ABA, GA_1_, GA_3_, GA_4_ in stems and over a time course of a year during which stems were experienced dormancy and dormancy release to growth and development; (**B**) correlation matrix between hormones and genes expression levels. The correlation values were calculated by spearman method and established by red (negative correlations) and blue (positive correlations) blocks. Stars above the numbers (*) means significant correlations.

Hormones like GAs, ABA, and IAA regulate plant development and dormancy, especially when the plants experience the change of climates. The growing cycle of *P. mume* stem occurs alongside seasonal changes, which may be affected by gene expression and hormonal content. Correlation assays were performed to explore the relationships between *PmDAMs*, *PmCBFs* and hormones. In February and August, IAA exhibited the highest and the lowest levels, respectively (Figure 5A). Both ABA and GA3 levels were significantly different at each stage of the growth cycle. ABA showed high levels in April and December, and the lowest in August. As for GA3, the highest level appeared in June with 10 or 100 times than that of GA1 or GA4.

The correlation matrix displayed the whole interaction among genes and hormones (Figure 5B). *PmCBF1–3* displayed an obvious negative correlation with *PmDAM1–3* and *PmCBF4–6* showed a significant positive correlation with *PmDAM4–6*. IAA negatively correlated with *PmDAM1–3*. In contrast, the other genes seem not to relate with IAA. GA3 was negatively controlled by two types of genes, especially *PmCBFs*. *PmCBF1–3* were positively correlated with the expression of ABA, and *PmDAM1–6* performed antipodally with ABA. The relative low values in correlation might infer the indirect regulation model for *PmCBFs* and *PmDAMs.*

### 2.6. Subcellular Localization Assessment

**Figure 6:**
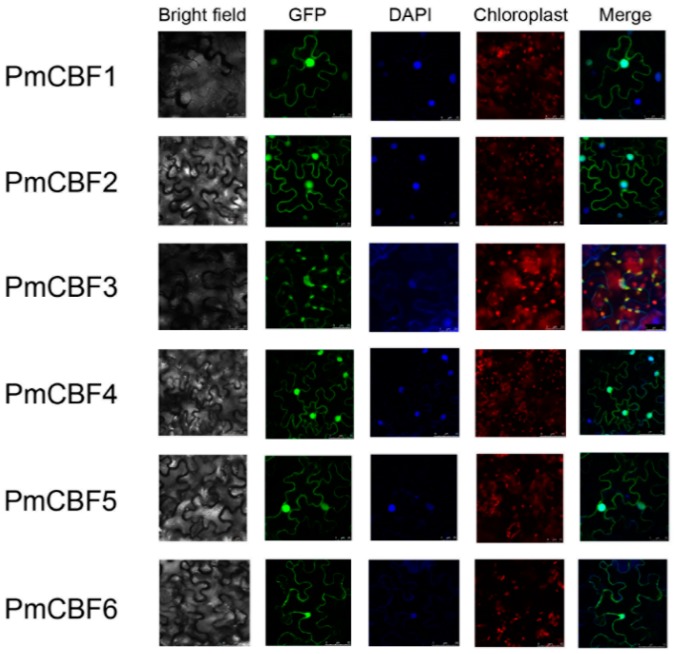
Subcellular localization of six PmCBFs. To determine the exact position of PmCBFs within the cell, subcellular localization experiments were performed using leaf tissues of *N. benthamiana*. The green fluorescent showed protein position. The blue fluorescent presented the nuclei position. The red fluorescent indicated the chloroplast position. The merge pictures of PmCBFs were formed by the pictures of GFP and DAPI except that PmCBF3 was formed by GFP, DAPI, and Chloroplast. For PmCBF1, 2, 3, and 5, the scale bar is 25; for PmCBF4 and 6, the scale bar is 50.

Controlled by a 35S promoter, the PmCBF with GFP was expressed in *N. benthamiana* leaves. All the PmCBFs were co-located alongside 4′,6-diamidino-2-phenylindole dihydrochloride (DAPI) in the parenchyma cells of leaves’ abaxial epidermis in *N. benthamiana*, suggesting that PmCBFs are primarily localized to the cell nucleus (Figure 6). PmCBF3 was also detected in chloroplasts.

### 2.7. Protein-Protein Interactions between CBF and DAM Genes in P. mume

Yeast two-hybrid assays were performed to determine protein complexes formed by PmCBFs and PmDAMs. PmCBF2, PmCBF3, PmCBF4, PmCBF5, and PmCBF6 had auto activation activity and cannot be used as baits. PmCBF1 and all PmDAMs were reconstructed in bait vectors. None of the experimental proteins had toxicity. Consequently, only the interaction profiles of PmCBF1 and six PmDAMs could be obtained by yeast two-hybrid assay.

**Figure 7:**
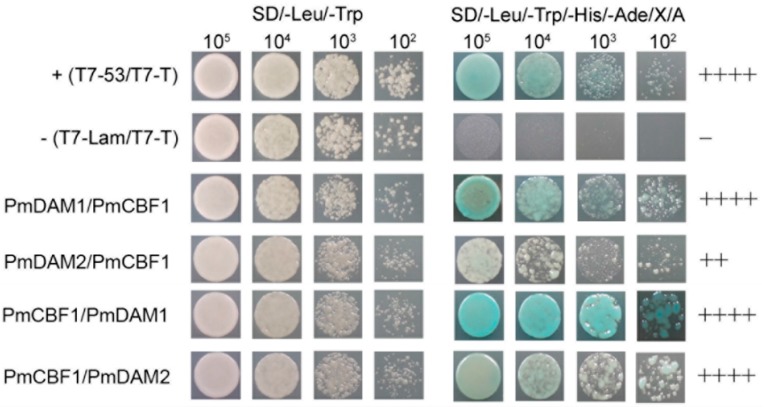
Protein-protein interactions between PmCBF1 and *PmDAMs*. T7–53/T7-T was positive control, and T7-Lam/T7-T was negative control. The symbol (+) was represented the capacity of the reaction. The more numbers of the symbol (+), stronger capacity of the reaction. The symbol (−) meant there were no interactions between proteins.

The dimerizations between PmCBF1 and PmDAMs are shown in Figure 7. PmDAM3, PmDAM4, and PmDAM5 could not form heterodimers with PmCBF1. PmDAM1 could dimerize strongly with PmCBF1, no matter used as a bait or a prey. While PmDAM2 and PmDAM6 showed poor interactions with PmCBF1. In addition, only acted as a bait, PmCBF1 interacted with PmDAM2, and PmDAM6 even stronger than the positive control.

### 2.8. BiFC Assay

**Figure 8:**
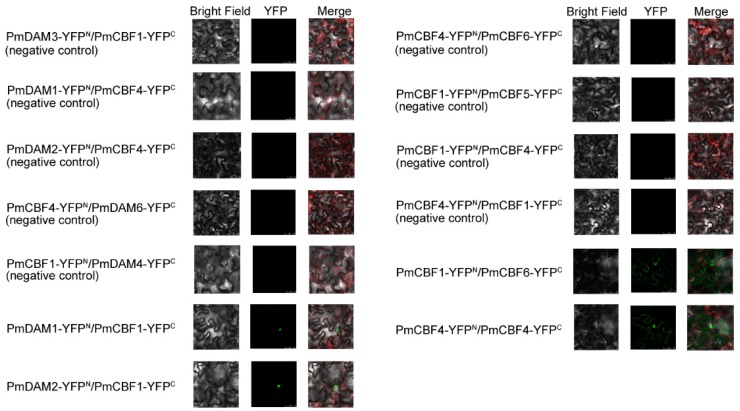
Bimolecular fluorescence complementation (BiFC) analysis of the protein interactions among *PmCBFs* and the reactions between *PmCBFs* and *PmDAMs*. Different combinations of the fused constructs were co-transformed into leaf cell of *N. benthamiana*, and then the cells were observed by confocal microscopy as described in “Materials and Methods”. In BiFC assays, a member of the same protein family can provide a negative control. The descriptions of the negative control tests were detail in “Results”. Bright field and YFP were excited at 514 nm. The green fluorescent presented protein position. The red fluorescent showed the chloroplast position.

Inter-protein interactions between PmCBF1 and PmDAMs and among PmCBFs were confirmed by BiFC assays using a separated yellow fluorescent protein (YFP). In each interaction, two proteins were fused with either C or N terminus of YFP designated as YFP^C^ or YFP^N^, respectively. There was no interaction in PmDAMs-YFP^C^/YFP^N^ or YFP^C^/PmDAMs-YFP^N^, and no interaction in PmCBFs-YFP^C^/YFP^N^ or YFP^C^/PmCBFs-YFP^N^ (Supplementary Figure S2). In *P. mume*, the *PmCBFs* are six homologous genes and the *PmDAMs* are six homologous genes as well. For *PmCBFs*, the protein sequences of PmCBF1, PmCBF3, and PmCBF4 are similar, while PmCBF5 and PmCBF6 have high homology. For *PmDAMs*, PmDAM1, PmDAM2, and PmDAM3 possess similar protein sequences, while PmDAM4, PmDAM5, and PmDAM6 have high homology. In BiFC assays, a member of the same protein family can provide a negative control. Thus, the interactions of PmDAM3-YFP^N^/PmCBF1-YFP^C^ and PmDAM1-YFP^N^/PmCBF4-YFP^C^ were used as the negative controls of PmDAM1-YFP^N^/PmCBF1-YFP^C^; the interactions of PmDAM3-YFP^N^/PmCBF1-YFP^C^ and PmDAM2-YFP^N^/PmCBF4-YFP^C^ were used as the negative controls of PmDAM2-YFP^N^/PmCBF1-YFP^C^; the interactions of PmCBF4-YFP^N^/PmDAM6-YFP^C^ and PmCBF1-YFP^N^/PmDAM4-YFP^C^ were used as the negative controls of PmCBF1-YFP^N^/PmDAM6-YFP^C^; the interactions of PmCBF4-YFP^N^/PmCBF6-YFP^C^ and PmCBF1-YFP^N^/PmCBF5-YFP^C^ were used as the negative controls of PmCBF1-YFP^N^/PmCBF6-YFP^C^, the interactions of PmCBF1-YFP^N^/PmCBF4-YFP^C^ and PmCBF4-YFP^N^/PmCBF1-YFP^C^ were used as the negative controls of PmCBF4-YFP^N^/PmCBF4-YFP^C^. The YFP signals were not detected in any negative control tests (Figure 8).

The protein-protein interactions between PmCBFs and PmDAMs were further verified through BiFC. The reactions (i.e., PmCBF1 and PmDAM1, PmCBF1 and PmDAM2) showed the YFP fluorescence in the nucleus, indicating that PmCBF1 could form dimers with PmDAM1, PmDAM2. The localization of YFP fluorescence in the nucleus revealed the significant interactive trend among PmCBFs. For PmCBFs, PmCBF4 could form a homodimer, and PmCBF1 could dimerize with PmCBF6 (Figure 8).

## 3. Discussion

### 3.1. CBF Genes in P. mume and Their Evolution

*AtCBF1–3* are located in a tandem array and show similar functions in cold response pathways. In *P. mume*, there are six *CBF* genes. Similar to *P. persica*, several of them are located in tandem arrays [29]. Each *PmCBFs* contain an AP2/ERF domain comprised of 58 amino acids. In the middle regions of these protein sequences, a CMIII-1 motif is found conserved among different species. For many plants, CBF/DREB1 proteins have two conservative sequences, PKK/RPAGRxKFxETRHP and DSAWR, that are distributed in the left and right of the AP2/ERF domain [41]. These CMIII-3 sequences help to distinguish CBF/DREB1 from other genes in AP2/ERF family. In the C-terminals of CBF proteins, there are two conserved motifs (CMIII-2 and CMIII-4) [13], which are both located in *PmCBFs* and more weakly conserved than other motifs. In the phylogenetic tree, six *PmCBFs* might have undergone different evolutionary processes because of their different evolutionary branches (Figure 2). These PmCBFs were clustered with the CBF proteins from other *Prunus* plants, suggesting that *Prunus CBFs* might experience a similar evolutionary process.

However, all *PmCBFs* had bias relationships with *DDF1* and *DDF2* (*AtCBF5* and *AtCBF6*) in *Arabidopsis*. Overexpression of *AtCBF5* and *AtCBF6* can cause dwarfism and delayed flowering [25], resulting from the repression of GA biosynthesis by CBF. This may provide extra explanations for the function diversity, even infer that *CBF* genes may appear after the specie formation. 

### 3.2. Positive Response to the Coldness by PmCBFs and PmDAM4–6

The CBF cold-response pathway is conserved in plants and contributes to plant dormancy and flowering time in previous studies [30,42,43,44]. Indeed, when low temperature occurred in October, all the *PmCBFs* were actively expressed (Figure 4B). This also confirms the upstream role of *PmCBFs* responding to low temperature. In particular, the possible relationship of *CBF* genes to dormancy in plants is highlighted [45]. In *Arabidopsis thaliana*, *AtCBF1–3* play an essential role in cold acclimation [23]. Overexpression of a peach *CBF* gene (*PpCBF1*) in apple inhibits plant growth, induces leaf senescence, causes early bud set, and delays bud break [27]. In apple, *MdDAM1–4* show specific expressions in dormant buds and are highly expressed during bud dormancy induction [46]. Moreover, during dormancy induction, *PpCBF* in *Pyrus pyrifolia* can enhance the transcriptional activity of *PpDAM1* and *PpDAM3* [3]. 

However, considering annual expression levels, the pattern of *PmCBF1–3* corresponded negatively to *PmDAM1–3*. During the growing stage from May to September when the expressions of *PmCBFs* were depressed to relatively low levels, the *PmDAM1–3* seem to be released, showing peak expressions in this stage. Therefore, a hypothesis is generated that *PmDAM* genes might function in stem for its growth. However, there are still no evidence for the growth maintenance, further researches would be required to analyze the unusual results. 

### 3.3. The Interactive Roles of Major Hormones

Developmental transitions in deciduous trees rely on endogenous hormones and genes expression changes that are triggered by environmental factors [47]. It becomes clear that the CBF genes constitute a central node of hormone cross-talk during cold stress response [48]. So far, it has been demonstrated that CBF expression is modulated by gibberellins, ABA, jasmonate, etc. For example, in trees, gibberellin s are particularly important because they may establish dormancy to help the plants avoid injuries due to cold. In the previous studies, the accumulation of CBF reduces bioactive GA to benefit the growth restraint by affecting key enzymes in the GA pathway [49]. In the stem growing stage of *P. mume*, GA3 owns a content more than 100 times than that of GA1 or GA4. The negative influence on GA3 also explains the function of CBF by the side. On the other hand, GA3 may be the major valid member in the growth of the stem by growth repressing DELLA proteins [50,51]. Jasmonate functions in the upstream of CBFs and positively regulate Arabidopsis freezing tolerance [52]. However, transcriptome of alfalfa shows us an overview that in response to freezing stress, transcripts of JAZ are up-regulated, which interact physically and repress the transcriptional function of ICE (inducer of CBF expresion) [53]. Further investigation may provide more functional mechanism for jasmonate. Additionally, ABA is produced by plants under cold conditions and plays an important role in mediating stress tolerance [54]. Recent studies report that ABA activates the CBF and corresponding genes in this pathway [55]. Interestingly, there exists a feedback regulation between PpDAM1 and ABA via upstream promoter binding sites, CArG for PpNCED3 and ABRE motifs for PpDAM1 [56]. Our results are also consistent with the previous confirmation that a positive correlation appear between *PmCBF1–3* and ABA in the stem, though more complex controls may exist in the downstream of PmCBFs.

In *P. mume*, there exists a mixed correlation for hormones around the *PmCBFs*, shown in Figure 5. There is another interesting observation in that *PmDAM1–3* shows high correlation with IAA. The special expression patterns drive us to speculate the involvement of *PmDAM1–3* in growth induction. But further studies are needed to decode the other identities of *PmDAM1–3.*

### 3.4. Proposed Regulation Network between Hormones and PmCBF and PmDAMs in Stem Cold Response

**Figure 9:**
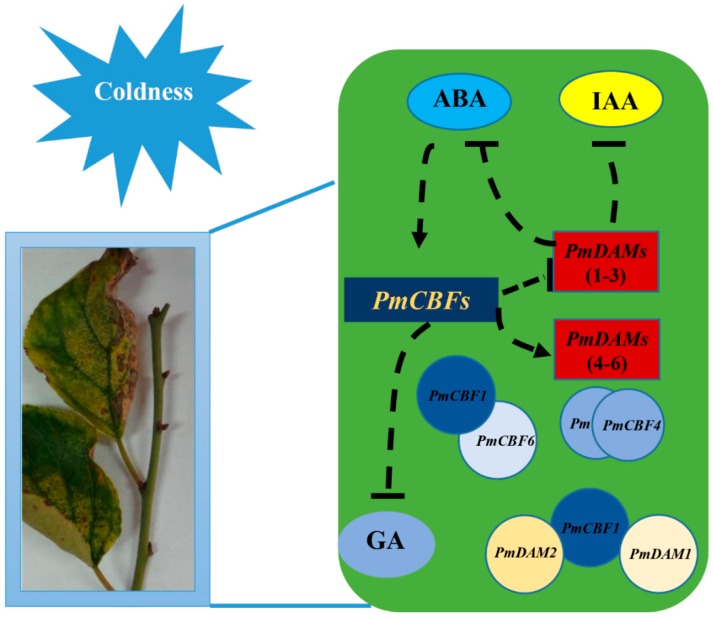
Schematic representation of molecular regulation model of hormones, *PmCBFs*, and *PmDAMs* during stem growth and dormancy. PmCBF1 could make heterogeneous dimers with PmDAM1, PmDAM2, and PmCBF6. PmCBF4 formed homologous dimers. The dotted arrows mean potential positive regulations. And the dotted crosses mean potential negative regulations.

The present studies have shown that *DAM* genes have relationships with plant dormancy induction and release [36,57,58,59]. During dormancy process, *PmCBF1–3* display negative correlation with *PmDAM1–3* and *PmCBF4–6* establish opposite roles with *PmDAM4–6*. The expressions of *CBF* genes can be induced by low temperatures [19,27]. In soybeans, most *GmDREB1s* are strongly influenced by various abiotic stresses, including low temperatures, high salt contents, drought, and heat [60]. The expressions of *CBF* genes in plants are associated with not only abiotic stresses but also hormones. *PnDREB1* in *Papaver nudicaule* is involved in both GA and abiotic stresses pathways [61]. Furthermore, *SwDREB1* from sweet potato participates in the ABA-independent pathway [62]. In this case, five PmCBFs are primarily expressed in the cell nucleus, which create possibilities for protein interactions among these proteins. Taken together, a hypothesis of molecular model for hormones, *PmCBFs*, and *PmDAMs* during the stem-growth process was proposed (Figure 9). For *P. mume*, six *PmCBFs* and *PmDAM4–6* join in the regulation of cold-response and dormancy actively, and formed different types of protein complexes, especially PmCBF1-PmDAM1 and PmCBF1-PmDAM2. However, it remains unclear why CBFs and DAMs has to function as homodimers or heterodimers. The research in Arabidopsis demonstrate AtCBFs functioning as vital signal proteins link multiple pathway against abiotic stress [63]. The loss-function mutants of AtCBF1–3 created by CRISPER system also display the major function of AtCBFs in cold acclimation and freezing tolerance, even in salt tolerance and seedling development [64]. This throw a light in the exploration of genes that the regulation mechanisms seem different with Arabidopsis.

In the present study, the patterns of expression for both hormones and genes were analyzed to increase knowledge of cold-induced stem dormancy in this woody plant. Additionally, we introduced the inner interaction mode between PmCBFs and PmDAMs. This discovery may provide new insights into the roles that *PmCBFs*, *PmDAMs* and hormones play in regulating tree dormancy and growth. 

## 4. Material and Methods

### 4.1. Plant Materials

*P. mume* “Sanlun Yudie” was selected from Beijing Forestry University, Beijing, China (40°00′ N, 116°18′ E). All three independent individuals for sampling were grown under similar environments. Each biological repeat was mixed from same organs or tissues, and separated into three tubes to conduct technical repeats. The flower buds were collected to clone genes. The samples of seven different organs were obtained to study expression patterns of *PmCBFs*, including flower bud (10 October 2015), leaf bud (10 October 2015), flower (full blooming; 22 March 2015), leaf (10 May 2015), fruit (10 June 2015), seed (10 June 2015) and stem (10 October 2015). The upper halves of the stems during different periods (from 10 January 2015 to 10 December 2015) were sampled to examine the expressions of *PmCBFs* and *PmDAMs*. These stem samples included bark tissues and vascular cambium and were taken to extract RNA and phytohormone using liquid nitrogen.

### 4.2. Identification and Cloning of CBF Genes

*PmCBF* (GeneBank: JX846908.1) was used as a query to identify additional *CBFs* in the *P. mume* protein database (http://prunusmumegenome.bjfu.edu.cn/) with an *e*-value set to 1e-3 by blastp. Seven *PmCBFs* were obtained, but one lacked the AP2/ERF domain, according to a domain analysis of these genes in InterPro (http://www.ebi.ac.uk/interpro/). Consequently, six *CBF* genes in *P. mume* were identified (Supplementary Table S1), and specific primers were designed based on CDS sequences (Supplementary Data S1). To clone these *PmCBFs*, total RNA was isolated from flower buds with TRIzol reagent (Aidlab, Beijing, China) following the manufacturer’s instructions. To synthesize first-strand cDNA, 2 µg of total RNA was used with TIANScript First Strand cDNA Synthesis Kit (Tiangen, Beijing, China). PCR reactions using Prime STAR HS DNA polymerase (Takara, Dalian, China) were performed to obtain cDNA of *PmCBFs* and *PmDAMs*. A 50 μL mix was used in these reactions, containing 10 μL of 5 × Prime STAR buffer, 4 μL of dNTP (10 mM), 2 μL of each primer (10 μM, Supplementary Table S2), 0.5 μL of cDNA, and 0.5 μL of Prime STAR polymerase. These PCR reactions were fulfilled in the following conditions: 94 °C for 2 min; 30 cycles of 98 °C for 10 s, annealing temperature (Supplementary Table S2) for 5 s, and 72 °C for 1 min; 72 °C for 5 min; and holding at 4 °C. To recover fragments, Gel Extraction Kit (Biomiga, San Diego, CA, USA) was employed. And then these fragments were cloned into pMDTM18-T vectors (TaKaRa, China) and transformed into DH5α (Tiangen, China). After sequencing the PCR-positive colonies (Taihe, Beijing, China), the cDNA sequences of *PmCBFs* (Supplementary Data S2) were obtained. The plasmids of these genes were extracted using Plasmid Miniprep Kit I (Biomiga, San Diego, CA, USA). 

### 4.3. Phylogenetic Analyses

To execute multiple sequence alignment, the CBF protein sequences of *P. mume* and other species (one CBF protein each from *Prunus persica*, *Prunus davidiana*, *Prunus ferganensis*, *Prunus avium*, *Eucalyptus globulus*, *Hippophae rhamnoides*, and *Citrus jambhiri*, three *Malus domestica* CBF proteins, four CBF proteins each from *Nicotiana tabacum* and *Vitis riparia*, five *Populus trichocarpa* CBF proteins, and six *Arabidopsis thaliana* CBF proteins) were exploited using DNAMAN 7.0 software (Lynnon Corp., Quebec, QC, anada) by default parameters. To construct a phylogenetic tree of these CBF proteins (Supplementary Data S3), MEGA7.1 software was performed with the ML method. For this tree, the parameters were set to default and the bootstrap values were set to 1000.

### 4.4. RT-qPCR

PikoReal real-time PCR system (Thermo Fisher Scientific, Darmstadt, Germany) was used to conduct the RT-qPCR experiments with a 10 μL mix, including 5 μL of SYBR Premix ExTaqII (Takara, Dalian, China), 0.5 μL of each primer (10 μM, Supplementary Table S3), and 2 μL of cDNA (30 times dilution of 2 μg cDNA). *PmPP2A* was regarded as the reference gene [65], and the 2^–ΔΔ*C*t^ method was used to calculate relative expression levels. Three biological replicates (each including three technical replicates) were performed to calculate the standard deviation. The correlations between gene expressions and hormones were done by Spearman method, and significant was analysed with kruskal-wallis test in R.

### 4.5. Subcellular Localization Assessments

The sequences coding for *PmCBFs* were cloned into supper1300-GFP plasmid using specific primers (Supplementary Table S4) and In-Fusion HD Cloning Kit System (Clonetech, Mountain View, CA, USA) to obtain 35S::PmCBF::GFP fusion. The GFP construct (supper1300-PmCBF), checked after sequencing, was transferred to *Nicotiana benthamiana* following agroinfiltration. These plasmids were transformed into GV3101, *Agrobacterium tumefaciens* strains, followed by culturing on LB medium bearing rifampicin (50 μg/mL), kanamycin (50 μg/mL) and gentamicin (50 μg/mL) at 28 °C. Agrobacteria were harvested and suspended twice in an infiltration buffer with 150 µM acetosyringone, 10 mM MES and 10 mM MgCl_2_. Bacterial suspension culture concentrations were measured using spectroscopy at an optical density of 600 nm and the concentrations were adjusted at 0.5–0.8. The mixture was maintained at room temperature for 2–3 h in the dark. Using syringes, the bacterial culture was infiltrated on the abaxial surfaces of the leaves and sampling was performed after two days for location assessment. Nuclear position was precisely assessed by incubating *N. benthamiana* leaf tissues with the nucleus marker DAPI. Finally, infiltrated leaves were observed under Leica TCS SP8 Confocal Laser Scanning Platform (excitation/emission settings: 405 nm for DAPI and 488 nm for GFP).

### 4.6. Yeast Two-Hybrid Assays

Full-length cDNA sequences of six *P. mume CBF* genes and six *P. mume DAM* genes were amplified by PCR experiments with specific primers (Supplementary Table S5). In-Fusion HD Cloning Kit System (Clonetech, Mountain View, CA, USA) was utilised to clone these sequences into pGBKT7 vectors (bait; Clonetech, Mountain View, CA, USA) and pGADT7 vectors (prey; Clonetech, Mountain View, CA, USA) at the EcoRI and BamHI sites, respectively. The Yeastmaker Yeast Transformation System 2 (Clonetech, Mountain View, CA, USA) was used to transform the bait plasmids into Y2H gold yeast strains (Clonetech, Mountain View, CA, USA) and the prey plasmids into Y187 yeast strains (Clonetech, Mountain View, CA, USA), before culturing on SD/-Trp plates and SD/-Leu plates, respectively. Single colonies of each transformant were cultured for 16 h (30 °C, 250 rpm). For subsequent interactive tests, auto activation and toxicity of bait strains were tested, and two selective yeast strains (bait and prey) were mated using YPDA liquid medium (Clonetech, Mountain View, CA, USA) for 20 h (30 °C, 80 rpm). After mating, the diploid yeast strains, which had a cloverleaf structure observed by 40 × microscope, were cultured on DDO (SD/-Trp/-Leu) solid medium at 30 °C for 3–5 days. The single colonies were continually cultured in DDO liquid medium for 20–24 h (30 °C, 250 rpm). Then, these samples were centrifuged to obtain the yeast strains, 700 g for 2 min. The supernatant liquid was discarded. Subsequently, 1.5 mL aseptic ddH_2_O was added to suspended strains, and the previous centrifugal work was repeated. To ensure the OD_600_ values of yeast strains were approximately 0.8, the appropriate aseptic ddH_2_O was added. Finally, 100 μL of these yeast samples (1, 1/10, 1/100, and 1/1000) was severally cultured at 30 °C for 3–5 days using DDO solid medium and SD/-Leu/-Trp/-His/-Ade/X-α-Gal/Aba plates. The analysis of protein-protein interactions was viewed in triplicate.

### 4.7. BiFC Assays

Pair-wise cloning of the full-length cDNA sequences of *PmCBFs* into pCambia1300-YFP-C and pCambia1300-YFP-N was performed to obtain BiFC constructs. Co-expression was executed on *N. benthamiana* leaves, as described in subcellular localization assessments. Chimeric fluorescence from expressed fusion proteins was observed 2–3 days post-infiltration using Leica TCS SP8 Confocal Laser Scanning Platform (YFPs excited at 514 nm). Specific primers were used for BiFC assays (Supplementary Table S6).

### 4.8. Phytohormone Assays

IAA, GAs and ABA were quantifled from 100 mg samples from the stems of *P. mume* ‘Sanlun Yudie’ in February, April, June, August, October, and December, which were same in gene expression analyses. Every test had been taken in three biological replicates and flash frozen in liquid nitrogen and stored at −80 °C until analysis. Stems (80–100 mg) were ground and extracted overnight at 4 °C with 2 mL of 99:1 isopropanol–acetic acid with 50 ng of d2-GA1, d2-GA3, d2-GA4, d5-IAA, and d6-ABAadded as internal standards. Hormone determination was performed using high-performance liquid chromatography–mass spectrometry (HPLC-MS) analysis of citrate-buffered acetone extracts as described previously. Then each plant hormone underwent quantitative analysis. The molar amount of plant hormone = (signal of plant hormone × the molar amount of corresponding internal standard) × (correction factor)/(signal of corresponding internal standard in the same sample). 

## Supplementary Materials

Supplementary materials can be found at http://www.mdpi.com/1422-0067/19/2/15/s1.
