# Supplementary material for: Crosstalk of PmCBFs and PmDAMs Based on the Changes of Phytohormones under Seasonal Cold Stress in the Stem of Prunus mume
Source: Int J Mol Sci. 2018 Jan 23;19(2):15. doi: 10.3390/ijms19020015 (PMC5855539; doi:10.3390/ijms19020015)
Supplement: Supplementary file 1 [file ijms-19-00015-s001.pdf]

Supplementary Figures

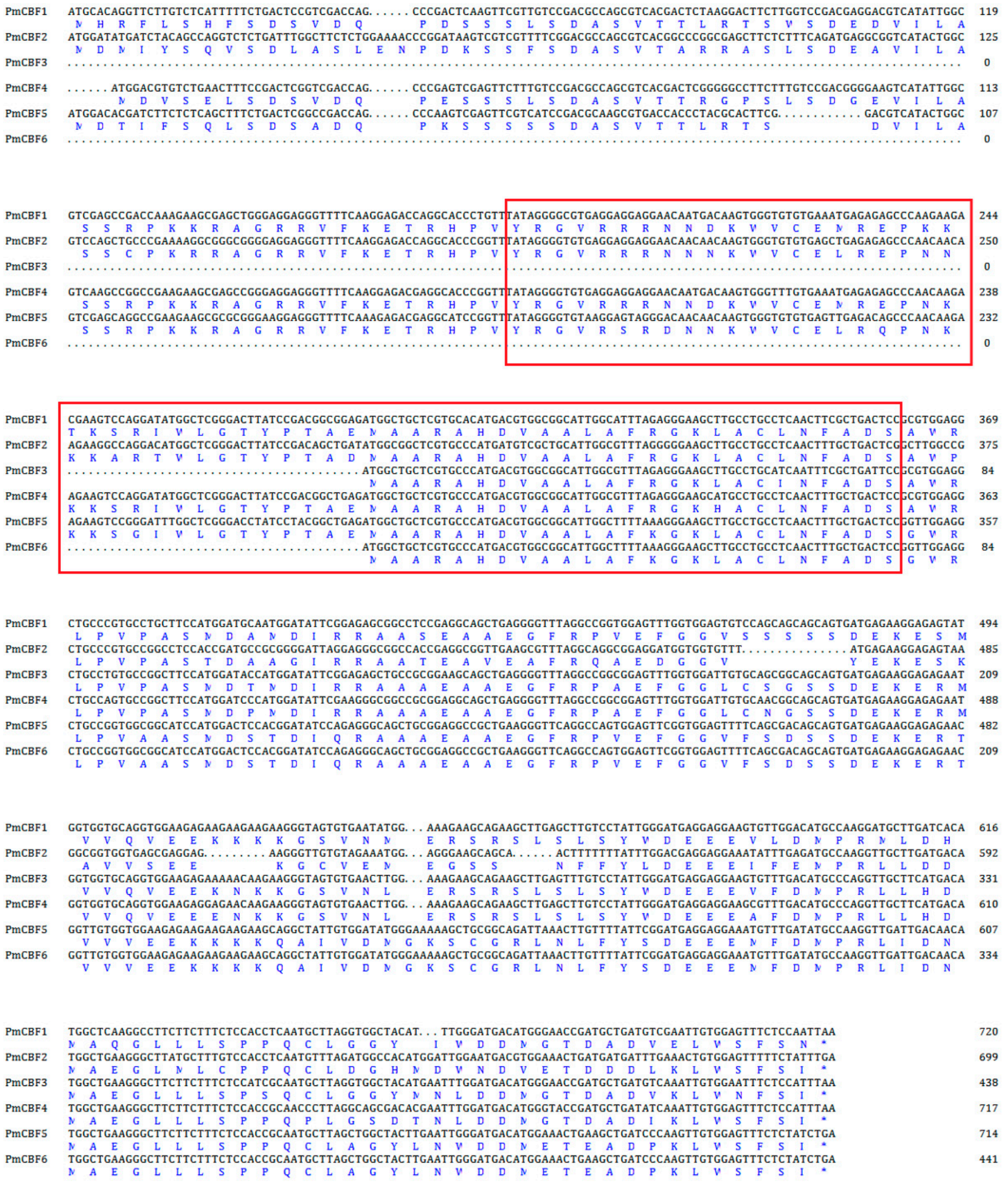

Supplementary Figure S1. The CDS and corresponding amino acid sequences of *PmCBFs*. The AP2/ERF domain was indicated in red rectangular region.

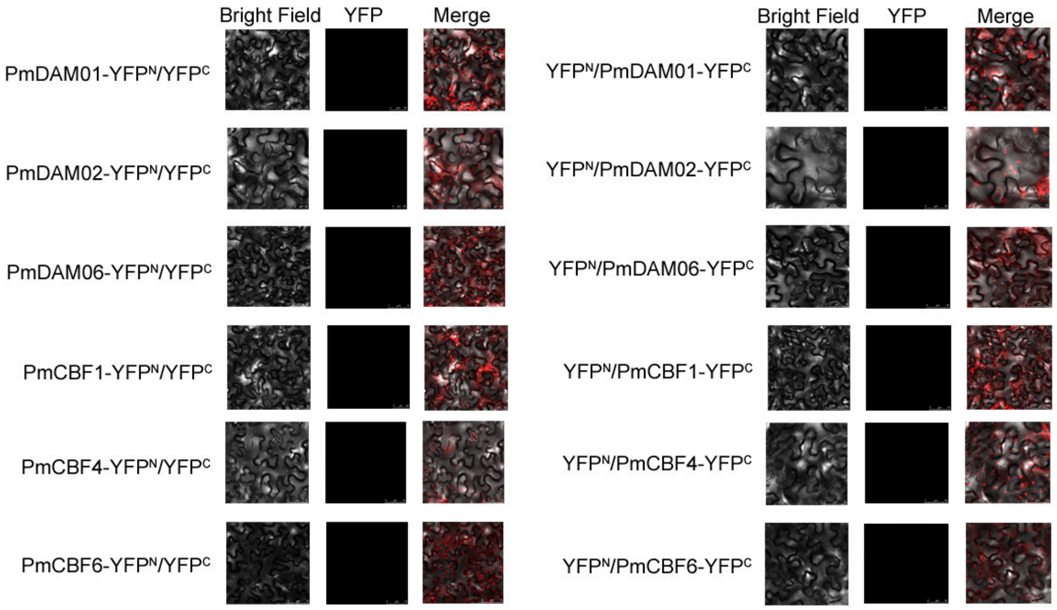

Supplementary Figure S2. Control experiments of BiFC assays. In every interactions, the two proteins were fused with either the C or N terminus of yellow fluorescent protein (YFP; designated as YFP^C or YFP^N, respectively). Different combinations of the fused constructs were co-transformed into leaf cell of *N. benthamiana*, and then the cells were observed by confocal microscopy. There was no interaction between YFP^N/YFP^C and PmDAMs-YFP^C/PmDAMs-YFP^N and no interaction between YFP^N/YFP^C and PmCBFs-YFP^C/PmCBFs-YFP^N. Bright field and YFP were excited at 514 nm. The red fluorescent showed the chloroplast position.

Supplementary Tables

Supplementary Table S1. *PmCBFs* identified in *P. mune* genome database.

Name	Gene ID	Length	locus
PmCBF1	Pm023769	987	Pa7:8068777:8070753:+
PmCBF2	Pm023772	678	Pa7:8098394:8099071:+
PmCBF3	Pm023773	438	Pa7:8100584:8101021:+
PmCBF4	Pm023775	717	Pa7:8104198:8104914:+
PmCBF5	Pm023777	795	Pa7:8114725:8115519:+
PmCBF6	Pm027913	441	C5001875:1668:2108:-

Supplementary Table S2. Primers used for cloning.

Gene	Forward primer	Reverse primer	Annealing temperatur e

PmCBF1	5' ATGCACAGGTTCTTGTCTGAT 3'	5' TTAATTGGAGAAACTCCACAA 3'	56 ℃
PmCBF2	5' ATGGATATGATCTACAGCCAG 3'	5' TCAAATAGAAAAACTCCACAG 3'	53 ℃
PmCBF3	5' ATGGCTGCTCGTGCCCATGAC 3'	5' TTAAATGGAGAAATTCCACAATTTGA 3'	64 ℃
PmCBF4	5' ATGGACGTGTCTGAACTTTCC 3'	5' TTAAATGGAGAAACTCCACAA 3'	57 ℃
PmCBF5	5' ATGGACACGATCTTCTCTCAG 3'	5' TCAGATAGAGAAACTCCACAA 3'	56 ℃
PmCBF6	5' ATGGCTGCTCGTGCCCATGAC 3'	5' TCAGATAGAGAAACTCCACAA 3'	64 ℃
PmDAM01	5'ATGAAAATGATGAGGGAGAAG3'	5' TTATGGAAGCCCCAGTTTGAG3'	52 ℃
PmDAM02	5'ATGGTGAAGACGATGAGGAAG3'	5' TTAGGGAAGCCCCAGTTTGAG3'	56 ℃
PmDAM03	5'ATGATGAGGAAGAAGATCAAG3'	5' TTAAGGAAGCCCCAGTTCGAG3'	51 ℃
PmDAM04	5'ATGGTGAAAATGATGAGGGAG3'	5' TTAGGAACGCCCCAGTTTGAG3'	54 ℃
PmDAM05	5'ATGATGAATAAGATCAAGATC3'	5' TTAACGCCCCAGTTTGAGAGA3'	47 ℃
PmDAM06	5'ATGGTGAAAATGATGAGGGAG3'	5' CTAGGGAAGCCCCAGTTTGAG3'	59 ℃

Supplementary Table S3. Primer used for real-time quantitative RT-PCR.

Gene	Forward primer	Reverse primer
PmPP2A	5'AGGGTTCGGCTCGCAATAATAGA3'	5'TGTTAGCAGCAGCATCACGAAT3'
PmCBF1	5' ATGCACAGGTTCTTGTCTGAT 3'	5' CGTCGGACCAAGAAGTCCTTA 3'
PmCBF2	5' AAAACCCGGATAAGTCGTCGT 3'	5' ATGTCCTGGCCTTCTTGTTGT 3'
PmCBF3	5' TGCATCAATTTCGCTGATTCC 3'	5' CGCTGCACACTCCACCAAATT 3'
PmCBF4	5' ACGTGTCTGAACTTTCCGACT 3'	5' GCTTGACGCCAATATGACTTC 3'
PmCBF5	5' TTCTCTCAGCTTTCTGACTCG 3'	5' AACCGGATGCCTCGTCTCTTT 3'
PmCBF6	5' TCCGGTTGGAGGCTGCCGGTG 3'	5' TTCCCATATCTACAATAGCCT 3'
PmDAM01	5'AGTATGAAGGATGTTATTCAA3'	5'CTTAAGTTCCTTGCTCAATCT3'
PmDAM02	5'AACCAGCTACGGCAGAGGATG3'	5'AGATTCAGATGACATGCCTT3'
PmDAM03	5'TCGGATTGAGCAAGGAACTGG3'	5'CATTCTCAGTTCTTCCTTTGT3'
PmDAM04	5'ACCCTTGTCCGTGTGATGGAA3'	5'ATCACCATCTGATTGTTGCCT3'
PmDAM05	5'AGGCTGAATAATAATATTGAA3'	5'TTAACGCCCCAGTTTGAGAGA3'
PmDAM06	5'AACCAACAACCAGTTAAGGCATA3'	5'CAATTACGGCAGATTCAGATGA3'

Supplementary Table S4. Primer used in PCR reactions for subcellular localization assessments.

Gene	Sequence 5′-3′
1300-PmCBF1-F	AGGGGCCCGGGGTCGACATGAAAATGATGAGGGAGAAG
1300-PmCBF1-R	GGTACCGGATCCACTAGTTGGAAGCCCCAGTTTGAG
1300-PmCBF2-F	AGGGGCCCGGGGTCGACATGGTGAAGACGATGAGGAAG
1300-PmCBF2-R	GGTACCGGATCCACTAGTGGGAAGCCCCAGTTTGAG
1300-PmCBF3-F	AGGGGCCCGGGGTCGACATGATGAGGAAGAAGATCAAG
1300-PmCBF3-R	GGTACCGGATCCACTAGTAGGAAGCCCCAGTTCGAG
1300-PmCBF4-F	AGGGGCCCGGGGTCGACATGGTGAAAATGATGAGGGAG
1300-PmCBF4-R	GGTACCGGATCCACTAGTGGAACGCCCCAGTTTGAG
1300-PmCBF5-F	AGGGGCCCGGGGTCGACATGATGAATAAGATCAAGATC
1300-PmCBF5-R	GGTACCGGATCCACTAGTACGCCCCAGTTTGAGAGA
1300-PmCBF6-F	AGGGGCCCGGGGTCGACATGGTGAAAATGATGAGGGAG
1300-PmCBF6-R	GGTACCGGATCCACTAGTGGGAAGCCCCAGTTTGAG

Supplementary Table S5. Primer used in PCR reactions for yeast two-hybrid assays.

Gene	Sequence 5′-3′	vector
BK-PmCBF1-F	CATGGAGGCCGAATTCATGCACAGGTTCTTGTCTGAT	pGBKT7
BK-PmCBF1-R	GCAGGTCGACGGATCCTTAATTGGAGAAACTCCACAA	
BK-PmDAM01-F	CATGGAGGCCGAATTCATGAAAATGATGAGGGAGAAG	pGBKT7
BK-PmDAM01-R	GCAGGTCGACGGATCCTTATGGAAGCCCCAGTTTGAG	
BK-PmDAM02-F	CATGGAGGCCGAATTCATGGTGAAGACGATGAGGAAG	pGBKT7
BK-PmDAM02-R	GCAGGTCGACGGATCCTTAGGGAAGCCCCAGTTTGAG	
BK-PmDAM03-F	CATGGAGGCCGAATTCATGATGAGGAAGAAGATCAAG	pGBKT7
BK-PmDAM03-R	GCAGGTCGACGGATCCTTAAGGAAGCCCCAGTTCGAG	
BK-PmDAM04-F	CATGGAGGCCGAATTCATGGTGAAAATGATGAGGGAG	pGBKT7
BK-PmDAM04-R	GCAGGTCGACGGATCCTTAGGAACGCCCCAGTTTGAG	
BK-PmDAM05-F	CATGGAGGCCGAATTCATGATGAATAAGATCAAGATC	pGBKT7
BK-PmDAM05-R	GCAGGTCGACGGATCCTTAACGCCCCAGTTTGAGAGA	
BK-PmDAM06-F	CATGGAGGCCGAATTCATGGTGAAAATGATGAGGGAG	pGBKT7
BK-PmDAM06-R	GCAGGTCGACGGATCCCTAGGGAAGCCCCAGTTTGAG	
AD-PmCBF1-F	GGAGGCCAGTGAATTCATGCACAGGTTCTTGTCTGAT	pGADT7
AD-PmCBF1-R	CGAGCTCGATGGATCCTTAATTGGAGAAACTCCACAA	
AD-PmDAM01-R	GGAGGCCAGTGAATTCATGAAAATGATGAGGGAGAAG	pGADT7
AD-PmDAM01-R	CGAGCTCGATGGATCCTTATGGAAGCCCCAGTTTGAG	
AD-PmDAM02-F	GGAGGCCAGTGAATTCATGGTGAAGACGATGAGGAAG	pGADT7
AD-PmDAM02-R	CGAGCTCGATGGATCCTTAGGGAAGCCCCAGTTTGAG	
AD-PmDAM03-F	GGAGGCCAGTGAATTCATGATGAGGAAGAAGATCAAG	pGADT7
AD-PmDAM03-R	CGAGCTCGATGGATCCTTAAGGAAGCCCCAGTTCGAG	
AD-PmDAM04-F	GGAGGCCAGTGAATTCATGGTGAAAATGATGAGGGAG	pGADT7
AD-PmDAM04-R	CGAGCTCGATGGATCCTTAGGAACGCCCCAGTTTGAG	
AD-PmDAM05-F	GGAGGCCAGTGAATTCATGATGAATAAGATCAAGATC	pGADT7

AD-PmDAM05-R	CGAGCTCGATGGATCCTTAACGCCCCAGTTTGAGAGA	
AD-PmDAM06-F	GGAGGCCAGTGAATTCATGGTGAAAATGATGAGGGAG	pGADT7
AD-PmDAM06-R	CGAGCTCGATGGATCCCTAGGGAAGCCCCAGTTTGAG	

Supplementary Table S6. Primer used in PCR reactions for BiFC assays.

Gene	Sequence 5′-3′
BiFC-PmCBF1-F	TGCAGGGAGGAGGATCCATGCACAGGTTCTTGTCTGAT
BiFC-PmCBF1-R	CGGTGCACTAGTGTCGACATTGGAGAAACTCCACAA
BiFC-PmDAM01-F	TGCAGGGAGGAGGATCCATGAAAATGATGAGGGAGAAG
BiFC-PmDAM01-R	CGGTGCACTAGTGTCGACTGGAAGCCCCAGTTTGAG
BiFC-PmDAM02-F	TGCAGGGAGGAGGATCCATGGTGAAGACGATGAGGAAG
BiFC-PmDAM02-R	CGGTGCACTAGTGTCGACGGGAAGCCCCAGTTTGAG
BiFC-PmDAM03-F	TGCAGGGAGGAGGATCCATGATGAGGAAGAAGATCAAG
BiFC-PmDAM03-R	CGGTGCACTAGTGTCGACAGGAAGCCCCAGTTCGAG
BiFC-PmDAM04-F	TGCAGGGAGGAGGATCCATGGTGAAAATGATGAGGGAG
BiFC-PmDAM04-R	CGGTGCACTAGTGTCGACGGAACGCCCCAGTTTGAG
BiFC-PmDAM05-F	TGCAGGGAGGAGGATCCATGATGAATAAGATCAAGATC
BiFC-PmDAM05-R	CGGTGCACTAGTGTCGACACGCCCCAGTTTGAGAGA
BiFC-PmDAM06-F	TGCAGGGAGGAGGATCCATGGTGAAAATGATGAGGGAG
BiFC-PmDAM06-R	CGGTGCACTAGTGTCGACGGGAAGCCCCAGTTTGAG

1 Supplementary Data

Supplementary Data S1. The CDS sequences of six *PmCBFs* identified in *P. mune* genome database.

>PmCBF1

ATGCACAGGTTCTTGTCTGATTTTTCTGACTCCGTCGACCAGCCCGACTCAAGTTCGTT

GTCCGACGCCAGCGTCACGACTCTAAGGACTTCTTGGTCCGACGAGGACGTCATATTG

GCGTCGAGCCGACCAAAGAAGCGAGCTGGGAGGAGGGTTTTCAAGGAGACCAGGCA

CCCTGTTTATAGGGGCGTGAGGAGGAGGAACAATGACAAGTGGGTGTGTGAAATGAG

AGAGCCCAAGAAGACGAAGTCCAGGATATGGCTCGGGACTTATCCGACGGCGGAGAT

GGCTGCTCGTGCACATGACGTGGCGGCATTGGCATTTAGAGGGAAGCTTGCCTGCCTC

AACTTCGCTGACTCCGCGTGGAGGCTGCCCGTGCCTGCTTCCATGGATGCAATGGATA

TTCGGAGAGCGGCCGCCGAGGCAGCTGAGGGGTTTAGGCCGGTGGAGTTTGGTGGAG

TGTCCAGCAGCAGCAGTGATGAGAAGGAGAGTATGGTGGTGCAGGTGGAAGAGAAG

AAGAAGAAGGGTAGTGTGAATATGGAAAGAAGCAGAAGCTTGAGCTTGTCCTATTGG

GATGAGGAGGAAGTGTTGGACATGCCAAGGTTGCTTGATCACATGGCTCAAGGCCTTC

TTCTTTCTCCACCTCAATGCTTAGGTGGCGACATTTGGGATGACATGGGAACCGATGCT

GATGTCGAATTGTGGATGATTGTGGAGTTTGGTGGAGTGTCCAGCAGCAGCAGTGATG

AGAAGGAGAGTATGGTGGTGCAGGTGGAAGAGAAGAAGAAGGGTAGTGTGAAT

ATGGAAAGAAGCAGAAGCTTGAGCTTGTCCTATTGGGATGAGGAGGAAGTGTTGGAC

ATGCCAAGGTTGCTTGATCACATGGCTCAAGGCCTTCTTCTTTCTCCACCTCAATGCTT

AGGTGGCGACATTTGGGATGACATGGGAACCGATGCTGATGTCGAATTGTGGAGTTTC

TCCAATTAA

>PmCBF2

ATGGATATGATCTACAGCCAGGTCTCTGATTTGGCTTCTCTGGAAAACCCGGATAAGTC

GTCGTTTTCGGACGCCAGCGTCACGGCCCGGCGAGCTTCTCTTTCAGATGAGGCGGTC

ATACTGGCGTCCAGCTGCCCGAAAAGGCGGGCGGGGAGGAGGGGTTTCAAGGAGAC

CAGGCACCCGGTTTATAGGGGTGTGAGGAGGAGGAACAACAACAAGTGGGTGTGTG

AGCTGAGAGAGCCCAACAACAAGAAGGCCAGGACATGGCTCGGGACTTATCCGACA

GCTGATATGGCGGCTCGTGCCCATGATGTCGCTGCATTGGCGTTTAGGGGGAAGCTTG

CCTGCCTCAACTTTGCTGACTCGGCTTGGCCGCTGCCCGTGCCGGCCTCCACCGATGC

CGCGGGGATTAGGAGGGCGGCCACCGAGGCGGAGGATGGTGGTGTTTATGAGAAGG

AGAGTAAGGCGGTGGTGAGCGAGGAGAAGGGTTGTGTAGAAATGGAGGGAAGCAGC

AACTTTTTTTATTTGGACGAGGAGGAAATATTTGAGATGCCAAGGTTGCTTGATGACAT

GGCTGAAGGGCTTATGCTTTGTCCACCTCAATGTTTAGATGGCCACATGGATTGGAATG

ACGTGGAAACTGATGATGATTTGAAACTGTGGAGTTTTTCTATTTGA

>PmCBF3

ATGGCTGCTCGTGCCCATGACGTGGCGGCATTGGCGTTTAGAGGGAAGCTTGCCTGCA

TCAATTTCGCTGATTCCGCGTGGAGGCTACCTGTGCCGGCTTCCATGGATACCATGGAT

ATTCGGAGAGCTGCCGCGGAAGCAGCGGAGGGGTTTAGGCCGGCGGAATTTGGTGGA

GTGTGCAGCGGCAGCAGTGATGAGAAGGAGAGAATGGTGGTGCAGGTGGAAGAGAA

GAACAAGAAGGGTAGTGTGAACTTGGAAAGAAGCAGAAGCTTGAGTTTGTCCTATTG

GGATGAGGAGGAAGTGTTTGACATGCCCAGGTTGCTTCATGACATGGCTGAAGGGCTT

CTTCTTTCTCCATCGCAATGCTTAGGTGGCTACATGAATTTGGATGACATGGGAACCGA

TGCTGATGTCAAATTGTGGAATTTCTCCATTTAA

>PmCBF4

ATGGACGTGTCTGAACTTTCCGACTCGGTCGACCAGCCCGAGTCGAGTTCTTTGTCCG

ACGCCAGCGTCACGACTCGGGGGCCTTCTTTGTCGGACGGGGAAGTCATATTGGCGTC

AAGCCGGCCGAAGAAGCGAGCCGGGAGGAGGGTTTTCAAGGAGACGAGGCACCCG

GTTTATAGGGGTGTGAGGAGGAGGAACAATGACAAGTGGGTTTGTGAAATGAGAGAG

CCCAACAAGAAGAAGTCCAGGATATGGCTCGGGACTTATCCGACGGCTGAGATGGCT

GCTCGTGCCCATGACGTGGCGGCATTGGCGTTTAGAGGGAAGCTTGCCTGCCTCAACT
TTGCTGACTCCGCGTGGAGGCTGCCAGTGCCGGCTTCCATGGATCCCATGGATATTCG
AAGGGCGGCCGCGGAGGCAGCTGAGGGGTTTAGGCCGGAGGAGTTTGGTGGAGTGT
GCAACGGCAGCAGTGATGAGAAGGAGAGAATGGTGGTGCAGGTGGAAGAGGAGAA
CAAGAAGGGTAGTGTGAACTTGGAAAGAAGCAGAAGCTTGAGCTTGTCCTATTGGGA
TGAGGAGGAAGCGTTTGACATGCCCAGGTTGCTTCATGACATGGCTGAAGGGCTTCTT
CTTTCTCCACCGCAACCCTTAGGCAGCGACACGAATTTGGATGACATGGGTACCGATG
CTGATATCAAATTGTGGAGTTTCTCCATTTAA

>PmCBF5

ATGGACACGATCTTCTCTCAGCTTTCTGACTCGGCCGACCAGCCCAAGTCGAGTTCGT
CATCCGACGCAAGCGTGACCACCCTACGCACTTCGGACGTCATACTGGCGTCGAGCA
GGCCGAAGAAGCGCGCGGGAAGGAGGGTTTTCAAAGAGACGAGGCATCCGGTTTAT
AGGGGTGTGAGGAGTAGGGACAACAACAAGTGGGTGTGTGAGTTGAGACAGCCCAA
CAAGAAGAAGTCCGGGATTTGGCTCGGGACCTATCCTACGGCTGAGATGGCTGCTCGT
GCCCATGACGTGGCGGCATTGGCTTTTAAAGGGAAGCTTGCCTGCCTCAACTTTGCTG
ACTCCGGTTGGAGGCTGCCGGTGGCGGCATCCATGGACTCCATGGATATCCAGAGGGC
AGCTGACGAGGGCGCCGGAGGGGTGCGGGCCGCGGGGCTGCCGGTGGCGGCATCCA
TGGACTCCATGGATATCCAGAGGGCAGCTGCGGAGGCCGCTGAAGGGTTCAGGCCAG
TGGAGTTCGGTGGAGTTTCCAGCGACAGCAGTGATGAGAAGGAGAGAACGGTTGTGG
TGGAAGAGAAGAAGAAGAAGCAGGCTATTGTAGATATGGGAAAAAGCTGCAGCAGA
TTAAACTTGTTTTATTCGGATGAGGAGGAAATGTTTGATATGCCAAGGTTGATTGACGA
CATGGCTGAAGGGCTTCTTCTTTCTCCACCCCAATGCTTAGCTGGCTACTTGAATTGGG
ATGACATGGAAACTGAATCTGATCCCAAGTTGTGGAGTTTCTCTATCTGA

>PmCBF6

ATGGCTGCTCGTGCCCATGACGTGGCGGCATTGGCTTTTAAAGGGAAGCTTGCCTGCC
TCAACTTTGCTGACTCCGGTTGGAGGCTGCCGGTGGCGGCATCCATGGACTCCATGGA
TATCCAGAGGGCAGCTGCGGAGGCCGCTGAAGGGTTCAGGCCAGTGGAGTTCGGTGG
AGTTTCCAGCGACAGCAGTGATGAGAAGGAGAGAACGGTTGTGGTGGAAGAGAAGA
AGAAGAAGCAGGCTATTGTAGATATGGGAAAAAGCTGCAGCAGATTAAACTTGTTTTA
TTCGGATGAGGAGGAAATGTTTGATATGCCAAGGTTGATTGACGACATGGCTGAAGGG
CTTCTTCTTTCTCCACCCCAATGCTTAGCTGGCTACTTGAATTGGGATGACATGGAAAC
TGAATCTGATCCCAAGTTGTGGAGTTTCTCTATCTGA

Supplementary Data S2. The cDNA sequences of six *PmCBFs* and six *PmDAMs* cloned from *P.*

mume ‘Sanlun Yudie’.

> PmCBF1

ATGCACAGGTTCTTGTCTCATTTTTCTGACTCCGTCGACCAGCCCGACTCAAGTTCGTT

GTCCGACGCCAGCGTCACGACTCTAAGGACTTCTTGGTCCGACGAGGACGTCATATTG

GCGTCGAGCCGACCAAAGAAGCGAGCTGGGAGGAGGGTTTTCAAGGAGACCAGGCA

CCCTGTTTATAGGGGCGTGAGGAGGAGGAACAATGACAAGTGGGTGTGTGAAATGAG

AGAGCCCAAGAAGACGAAGTCCAGGATATGGCTCGGGACTTATCCGACGGCGGAGAT

GGCTGCTCGTGCACATGACGTGGCGGCATTGGCATTTAGAGGGAAGCTTGCCTGCCTC

AACTTCGCTGACTCCGCGTGGAGGCTGCCCGTGCCTGCTTCCATGGATGCAATGGATA

TTCGGAGAGCGGCCTCCGAGGCAGCTGAGGGGTTTAGGCCGGTGGAGTTTGGTGGAG

TGTCCAGCAGCAGCAGTGATGAGAAGGAGAGTATGGTGGTGCAGGTGGAAGAGAAG

AAGAAGAAGGGTAGTGTGAATATGGAAAGAAGCAGAAGCTTGAGCTTGTCCTATTGG

GATGAGGAGGAAGTGTTGGACATGCCAAGGATGCTTGATCACATGGCTCAAGGCCTT

CTTCTTTCTCCACCTCAATGCTTAGGTGGCTACATTTGGGATGACATGGGAACCGATGC

TGATGTCGAATTGTGGAGTTTCTCCAATTAA

> PmCBF2

ATGGATATGATCTACAGCCAGGTCTCTGATTTGGCTTCTCTGGAAAACCCGGATAAGTC

GTCGTTTTCGGACGCCAGCGTCACGGCCCGGCGAGCTTCTCTTTCAGATGAGGCGGTC

ATACTGGCGTCCAGCTGCCCGAAAAGGCGGGCGGGGAGGAGGGTTTTCAAGGAGAC

CAGGCACCCGGTTTATAGGGGTGTGAGGAGGAGGAACAACAACAAGTGGGTGTGTG

AGCTGAGAGAGCCCAACAACAAGAAGGCCAGGACATGGCTCGGGACTTATCCGACA

GCTGATATGGCGGCTCGTGCCCATGATGTCGCTGCATTGGCGTTTAGGGGGAAGCTTG

CCTGCCTCAACTTTGCTGACTCGGCTTGGCCGCTGCCCGTGCCGGCCTCCACCGATGC

CGCGGGGATTAGGAGGGCGGCCACCGAGGCGGTTGAAGCGTTTAGGCAGGCGGAGG

ATGGTGGTGTTTATGAGAAGGAGAGTAAGGCGGTGGTGAGCGAGGAGAAGGGTTGTG

TAGAAATGGAGGGAAGCAGCAACTTTTTTTATTTGGACGAGGAGGAAATATTTGAGAT

GCCAAGGTTGCTTGATGACATGGCTGAAGGGCTTATGCTTTGTCCACCTCAATGTTTAG

ATGGCCACATGGATTGGAATGACGTGGAAACTGATGATGATTTGAAACTGTGGAGTTT

TTCTATTTGA

> PmCBF3

ATGGCTGCTCGTGCCCATGACGTGGCGGCATTGGCGTTTAGAGGGAAGCTTGCCTGCA

TCAATTTCGCTGATTCCGCGTGGAGGCTGCCTGTGCCGGCTTCCATGGATACCATGGAT

ATTCGGAGAGCTGCCGCGGAAGCAGCTGAGGGGTTTAGGCCGGCGGAGTTTGGTGGA

TTGTGCAGCGGCAGCAGTGATGAGAAGGAGAGAATGGTGGTGCAGGTGGAAGAGAA

AAACAAGAAGGGTAGTGTGAACTTGGAAAGAAGCAGAAGCTTGAGTTTGTCCTATTG

GGATGAGGAGGAAGTGTTTGACATGCCCAGGTTGCTTCATGACATGGCTGAAGGGCTT

CTTCTTTCTCCATCGCAATGCTTAGGTGGCTACATGAATTTGGATGACATGGGAACCGA

TGCTGATGTCAAATTGTGGAATTTCTCCATTTAA

> PmCBF4

ATGGACGTGTCTGAACTTTCCGACTCGGTCGACCAGCCCGAGTCGAGTTCTTTGTCCG

ACGCCAGCGTCACGACTCGGGGGCCTTCTTTGTCCGACGGGGAAGTCATATTGGCGTC

AAGCCGGCCGAAGAAGCGAGCCGGGAGGAGGGTTTTCAAGGAGACGAGGCACCCG

GTTTATAGGGGTGTGAGGAGGAGGAACAATGACAAGTGGGTTTGTGAAATGAGAGAG

CCCAACAAGAAGAAGTCCAGGATATGGCTCGGGACTTATCCGACGGCTGAGATGGCT

GCTCGTGCCCATGACGTGGCGGCATTGGCGTTTAGAGGGAAGCATGCCTGCCTCAACT

TTGCTGACTCCGCGTGGAGGCTGCCAGTGCCGGCTTCCATGGATCCCATGGATATTCG

AAGGGCGGCCGCGGAGGCAGCTGAGGGGTTTAGGCCGGCGGAGTTTGGTGGATTGTG

CAACGGCAGCAGTGATGAGAAGGAGAGAATGGTGGTGCAGGTGGAAGAGGAGAAC

AAGAAGGGTAGTGTGAACTTGGAAAGAAGCAGAAGCTTGAGCTTGTCCTATTGGGAT

GAGGAGGAAGCGTTTGACATGCCCAGGTTGCTTCATGACATGGCTGAAGGGCTTCTTC

TTTCTCCACCGCAACCCTTAGGCAGCGACACGAATTTGGATGACATGGGTACCGATGC

TGATATCAAATTGTGGAGTTTCTCCATTTAA

> PmCBF5

ATGGACACGATCTTCTCTCAGCTTTCTGACTCGGCCGACCAGCCCAAGTCGAGTTCGT

CATCCGACGCAAGCGTGACCACCCTACGCACTTCGGACGTCATACTGGCGTCGAGCA

GGCCGAAGAAGCGCGCGGGAAGGAGGGTTTTCAAAGAGACGAGGCATCCGGTTTAT

AGGGGTGTAAGGAGTAGGGACAACAACAAGTGGGTGTGTGAGTTGAGACAGCCCAA

CAAGAAGAAGTCCGGGATTTGGCTCGGGACCTATCCTACGGCTGAGATGGCTGCTCGT

GCCCATGACGTGGCGGCATTGGCTTTTAAAGGGAAGCTTGCCTGCCTCAACTTTGCTG

ACTCCGGTTGGAGGCTGCCGGTGGCGGCATCCATGGACTCCACGGATATCCAGAGGG

CAGCTGCGGAGGCCGCTGAAGGGTTCAGGCCAGTGGAGTTCGGTGGAGTTTTCAGCG

ACAGCAGTGATGAGAAGGAGAGAACGGTTGTGGTGGAAGAGAAGAAGAAGAAGCA

GGCTATTGTGGATATGGGAAAAAGCTGCGGCAGATTAAACTTGTTTTATTCGGATGAGG

AGGAAATGTTTGATATGCCAAGGTTGATTGACAACATGGCTGAAGGGCTTCTTCTTTCT

CCACCGCAATGCTTAGCTGGCTACTTGAATTGGGATGACATGGAAACTGAAGCTGATC

CCAAGTTGTGGAGTTTCTCTATCTGA

> PmCBF6

ATGGCTGCTCGTGCCCATGACGTGGCGGCATTGGCTTTTAAAGGGAAGCTTGCCTGCC

TCAACTTTGCTGACTCCGGTTGGAGGCTGCCGGTGGCGGCATCCATGGACTCCACGGA

TATCCAGAGGGCAGCTGCGGAGGCCGCTGAAGGGTTCAGGCCAGTGGAGTTCGGTGG

AGTTTTCAGCGACAGCAGTGATGAGAAGGAGAGAACGGTTGTGGTGGAAGAGAAGA

AGAAGAAGCAGGCTATTGTGGATATGGGAAAAAGCTGCGGCAGATTAAACTTGTTTTA

TTCGGATGAGGAGGAAATGTTTGATATGCCAAGGTTGATTGACAACATGGCTGAAGGG

CTTCTTCTTTCTCCACCGCAATGCTTAGCTGGCTACTTGAATTGGGATGACATGGAAAC

TGAAGCTGATCCCAAGTTGTGGAGTTTCTCTATCTGA

>PmDAM1
ATGAAAATGATGAGGGAGAAGATCAAGATCAAGAAGATTGACAACTTGCCTGCAAG
GCAAGTGACCTTCTCAAAGAGGAGGAGAGGGATCTTCAAGAAAGCTGCAGAGTTAT
CTGTTCTGTGTGAATCTGAGGTGGCAGTTGTCATCTTTTCTGCTACTGGCAAGCTTTTT
GATTATTCAAGCTCAAGTATGAAGGATGTTATTGAAAGGTACCAAGCGCACATAAAT
GGTGGTGAAAAATTTAACGAACGGTCTATTGAGTTGCAGCCAGAGTATGAAAACCAC
ATCAGATTGAGCAAGGAACTTAAGGAGAAGAGCCGCCAGCTGAGGCAGATGAAAGG
AGAGGATCTTGAAGAGCTGAATTTTGATGAGTTGCAGAAGTTAGAACAACTGGTGGA
TGCAAGCCTTGGCCGTGTGATTGAAACTAAGGACGAACGGATTATGAGTGAGATTAT
GGCACTTGAAAGAAAGAGAGCTGAGCTTGTAAAAGCCAACAAACAGCTAAGGCAGA
GGATGTTATTCAGAGGAAATATTGGACCTGAGCTTATGAAGCCGGAGAGGTTGAATA
ATAATTTTGGTGGTGGAGGAGAAGAAGAAGGTATGTCATCTGAATCTGCTACCTCCA
CCACCTGCAACAGTGCTCCGAGTCTCTCTCTTGAAGATGACTCCGACGACGTCACTTT
ATCTCTCAAACTGGGGCTTCCATAA
>PmDAM2
ATGGTGAAGACGATGAGGAAGAAGATCAAGATCAAGAAGATTGACTACTTGCCTGC
AAGGCAGGTGACCTTCTCAAAGAGGAGGAGAGGGATCTTCAAAAAAGCTGAGGAGC
TATCTGTTCTGTGTGAATCTGAGGTGGCAGTTGTCATCTTTTCTGCTACTGGCAAGCTT
TTTGATTATTCAAGCTCAAGTACAAAGAATGTTGTTGAAAGGTATAAAGCGCACACA
AATGGTGTCGAAAAATCGGACGAACTGTCTGTTGAGCTGCAGCTAGAAATTGAAAAC
CAGATCAGATTGAACAAGGAACTTGCGGAGAAGAGCCGCCAGCTGAGGCAGATGAG
AGGAGAGGATCTTGAAGAGCTGAATATTGATGAGTTGCAGAAGTTAGAACAACTGGT
GGAGGCAAGCCTTGGCCGTGTGATTGAAACTAAGGAAGAACTGATTATGAGTGAGAT
TATGGCACTTGAAAGAAAGGGAGCTGAGCTGGTAGAAGCCAACAACCAGCTACGGC
AGAGGATGGTGATGTTATCCAGAGGAAATATTGGACCTGGGCTTACGGAGCCGGAGA
GGTTCATTAATAATATTGGAGATGGAGGAGAAGAAGGCATGTCATCTGAATCTGCCA
CAAATGCAACCATCAGCAGCTGCAGCAGTGGTCTCAGTCTCTCTCTTGAAGATGACTG
CTCAGACGTCACTTTAGCTCTCAAACTGGGGCTTCCCTAA
>PmDAM3
ATGATGAGGAAGAAGATCAAGATCAAGAAGATTGATTGCTTGCCTGCAAGGCAGGT
GACTTTCTCAAAGAGGAGAAGAGGGATCTTCAAGAAAGCTGCAGAGCTATCTGTTCT
GTGTGAATCTAAGGTGGCAGTTGTCATATTTTCTGCTACTGGCAAGCTTTTTGATTATT
CAAGCTCAAGTATCAAGGATGTTATTGAAAGCTACAAAGCGCACAAAAATGGTGTCA
AAAAATCGGACGAACCGTCTGTTGAGCTACAGCTAGAGAATGAAAATCACATCGGA
TTGAGCAAGGAACTGGAGGAGAAGAGCCATCAGCTGAGGCAGATGAAAGCAGAGG

ATCTTGACGAGCTGAATTTTGATGAGTTGCAGAAGTTAGAACAACTGGTGGACACAA
GCCTTAGCCGTGTGATTGAAACAAAGGAAGAACTGAGAATGAGTGAGATTATGGCA
CTTGAAAGAAAGGGAGCTGAGCTGGTAGAAGCCAACAACCAGCTAAAGCAGACGAT
GGTGATGTTATCCGGAGGAAATACTGGACCTACGCTTATGGATCCGGAGAGGTTGAA
TGATAATGTCGGAGGTGGAGGAGAAGAAGAAGGCATGTCATCTGAATCTGCTATCTC
CACCACCTGCAACAGTGCTCTCAGTCTCTCCATTGGAGATGACTCCGACGACGTCACT
TTATCTCTCGAACTGGGGCTTCCTTAA
>PmDAM4
ATGATGAGGAAGAAGATCAAGATCAAGAAGATTGACTACCTGCCAGCAAGGCAGGT
GACATTCTCAAAGAGGAGAAGAGGGATCTTCAAGAAAGCTGCAGAGCTATCTGTTCT
GTGTGAATCTGAGGTGGCAGTTGTCATCTTTTCTGCTACTGGCAAGCTTTTTGATTATT
CAAGCTCAAGTATCAAGGATGTTATTGAAAGGTACAAAGCGCGCACAAATGGTGTCG
AAAAATCGGATGAACAGTCTCTTGAGCTGCAGCTGGAGAATGAAAACCGCATCAAA
CTCAGTACGGAACTCGAGGAGAAGAACCGCCAGCTGAGGCGGATGAAAGGTGAGGA
TCTTGAAGAGCTGGATCTGGATGAGTTGCTGAAGTTGGAACAACTGGTGGAAGCAAC
CCTTGTCCGTGTGATGGAAACTAAGGAAGAACTGATTATGAGTGATATTGTGGCACTT
GATAAAAAGGGAACTGAGCTGGTAGAAGGCAACAATCAGATGGTGATGTTAAGGGA
CAGGATGGTGATGTTATCCAAAAGAAGTACCGGACCTGCGCTTATGGAGCCATCTGA
CTCTGCTACCTCCACCAGCTGCAACAGTGCTCTGAGTCTTTCTCTTGAAGATGAATGCT
CCGACGACGCCATTTTATCTCTCGAACTGGGGCGTTCCTAA
>PmDAM5
ATGATGAATAAGATCAAGATCAAGAAGATTGACTACTTGCCTGCAAGGCAGGTGACC
TTCTCAAAAAGGAGAAGAGGGCTCTTCAAGAAAGCTGCAGAGCTATCTGTTCTGTGT
GAATCTGAGGTGGCAGTTGTCATCTTTTCTGCCACTGGCAAGCTTTTTGATTATTCAAG
CTCAAGTACCAAGGATGTTATTGAAAGGTACAACGCAGACATGAATGGTGTCGAAA
AATCGAACAATCAAGAGATTGAGCTGCAGCTGGAGAATGAAAACCACATCAAACTG
AGTAAGGAACTCGAGAAGACGAGCCACCAGCTGAGGCAGATGAAAGGTGAGGATCT
TGAAGGGCTGAATCTGGATGAGTTGCTGAAGTTGGAACAACTGGTGGAAGCAAGCCT
TGGCCGTGTCATGGAAACTAAGGAAGAGCTGATTAAGAGTGAGATTATGGAACTCGA
AAGAAAGGGAGCTGAGCTAGTTGAAGCCAACAGCCAGCTAAGGCAGACGATGGTGA
TGTTATCCGGAGGAAATACTGGACCTGCGCTTATGGATCCGGAGAGGCTGAATAATA
ATATTGAAGGTGGAGGAGAAGAAGAAGGCATGTCAGCTGAATCTGCTATCTCCACCA
CCTGCAACAGTGCTGTCAGTCTCTCTCTTGAAGATGACTCCTCCGATGAGGTCACTTT
GTCTCTCAAACTGGGGCGTTAA
>PmDAM6
ATGGTGAAAATGATGAGGGAGAAGATCAAGATCAAGAAGATTGACTACCTGCCAGC
AAGGCAGGTTACCTTTTCAAAGAGAAGAAGAGGGCTCTTCAAGAAAGCTGCAGAGC
TATCGGTTCTGTGTGAATCTGAGGTGGCTGTCGTCATCTTTTCTGCCACTGACAAGCTC
TTTCATTATTCAAGCTCAAGTACCGAGGATGTTATTGAAAGGTACAAAGCGCACACA
GGTGGTGCCGAAAAATCAGACAAACAGTTTCTTGAGCTGCAACTGGAGAATGAAAA
CAACATCAAACTGAGTAAGGAACTCGAGGAGAAGAGCCGCCAGCTGAGGCAGATGA
AAGGTGAGGATCTTGAAGGGCTGAATCTGGATGAGCTGCTGAAGTTAGAACAAGTGG

TGGAAGCAAGCCTTGGCCGTGTGATAGAAACTAAGGAAGAGCTGATTATGAGTGCGA
TTATGGCACTGGAGAAAAAGGGAGCTGAGCTGGTAGAAACCAACAACCAGTTAAGG
CATAGGATGGTGATGTTATCCGGAGGAAATACTGGACCTGCGTTTGTGGAGCCGGAG
ACGTTGATTACTAATGTTGGAGGTGGAGGACGAGAAGACGACATGTCATCTGAATCT
GCCGTAATTGCCACCTCCACCAGCTGCAACAGTGCTTTCAGTCTCTCTCTTGAAGATG
ACTGCTCCGATGTCACTTTATCTCTCAAACTGGGGCTTCCCTAG

Supplementary Data S3. The proteins sequences of CBF genes.

>PmCBF1
MHRFLSHFSDSVDQPDSSSLSDASVTTLRTSWSDEDVILASSRPKKRAGRRVFKETRHPVYR
GVRRRNNDKWVCEMREPKKTKSRIWLGTYPTAEMAARAHDVAALAFRGKLACLNFADS
AWRLPVPASMDAMDIRRAASEAAEGFRPVEFGGVSSSSSDEKESMVVQVEEKKKKGSVNM
ERSRSLSLSYWDEEEVLDMPRMLDHMAQGLLLSPPQCLGGYIWDDMGTDADVELWSFSN*
>PmCBF2
MDMIYSQVSDLASLENPDKSSFSDASVTARRASLSDEAVILASSCPKRRAGRRVFKETRHPV
YRGVRRRNNNKWVCELREPNNKKARTWLGTYPTADMAARAHDVAALAFRGKLACLNF
ADSAWPLPVPASTDAAGIRRAATEAVEAFRQAEDGGVYEKESKAVVSEEKGCVEMEGSSN
FFYLDEEEIFEMPRLLDDMAEGLMLCPPQCLDGHMDWNDVETDDDLKLWSFSI*
>PmCBF3
MAARAHDVAALAFRGKLACINFADSAWRLPVPASMDTMDIRRAAAEAAEGFRPAEFGG
LCSGSSDEKERMVVQVEEKNKKGSVNLERSRSLSLSYWDEEEVFDMPRLLHDMAEGLLLSP
SQCLGGYMNLDDMGTDADVKLWNFSI*
>PmCBF4
MDVSELSDSVDQPESSSLSDASVTTRGPSLSDGEVILASSRPKKRAGRRVFKETRHPVYRGVR
RRNNDKWVCEMREPNKKKSRIWLGTYPTAEMAARAHDVAALAFRGKHACLNFADSAW
RLPVPASMDPMDIRRAAAEAAEGFRPAEFGGLCNGSSDEKERMVVQVEEENKKGSVNLER
SRSLSLSYWDEEEAFDMPRLLHDMAEGLLLSPPQPLGSDTNLDDMGTDADIKLWSFSI*
>PmCBF5
MDTIFSQLSDSADQPKSSSSSDASVTTLRTSDVILASSRPKKRAGRRVFKETRHPVYRGVRSR
DNNKWVCELRQPNKKKSGIWLGTYPTAEMAARAHDVAALAFKGKLACLNFADSGWRL
PVAASMDSTDIQRAAAEAAEGFRPVEFGGVFSDSSDEKERTVVVEEKKKKQAIVDMGKSC
GRLNLFYSDEEEMFDMPRLIDNMAEGLLLSPPQCLAGYLNWDDMETEADPKLWSFSI*
>PmCBF6
MAARAHDVAALAFKGKLACLNFADSGWRLPVAASMDSTDIQRAAAEAAEGFRPVEFGG
VFSDSSDEKERTVVVEEKKKKQAIVDMGKSCGRLNLFYSDEEEMFDMPRLIDNMAEGLLLS
PPQCLAGYLNWDDMETEADPKLWSFSI*
>PpCBF1
MVMDMIFAQVSDSADQPRSSSSSDASVSTLRTSDVILASSSPKKRAGRKVFKETRHPVYRGV
RRRDNNKWVCELRQPNKKKSGIWLGTYPTAEMAARAHDVAALAFKGKLACLNFADSG
WRLPVAASMDSMDIQRAAAEAAEGFRPVEFGGVSSGSSDEKERMVVVEEKKKKQAIVDM
GKSCSRLNLFYLDEEEMFDMPRLIDNMAEGLLLSPPQCLAGYLNWDDMETEADSKLWSFS
I*

>PpCBF2
MDMFSAQLSDSPDQPESSSFSDASVTTLPASSSDENVILASSRPKKRAGRRVFKETRHPVYRG
VRRRNNNKWVCELREPNNKKSRIWLGTYPTAEMAARAHDVAALAFRGKLACINFADSA
WRLPLPASMDTMDIRRAAAEAAEGFRPAEFGGLSSGSSDEKEMNLSVDMEKNSSLCLFYL
DEEEMFDMPRLIDNMAQGLLLSPPQCSAGYLNWDDVETEADAKLWSFSI*
>PpCBF3
MDLSQLSDPVDQPESSSLSDASVTTQGPSWSDGDVILASSLPKKRAGRRVFKETRHPVYRGV
RRRNNDKWVCEMREPNKKKSRIWLGTYPTAEMAARAHDVAALAFRGKLACINFADSAW
RLPLPASMDTMDIRRAAAEAAERFRPAEFGGMCSGSSDEKERMVVQVEEEDKKGSVNLER
SLSLSCWDEEEAFDMPRLLHDMAEGLLLSPPQPLGSDMNLDDMGTDADIKLWSFSI*
>PpCBF4
MDLIYSQVSDLASLENPDTSSFWNASVTARRASLSVEEVILASSCPKRRAGRRVFKETRHPV
YRGVRRRNNDKWVCELRDPNNKKARIWLGTYPKAEMAARAHDVAALAFRGKLACLNF
ADSAWRLPVPASNDAAEIRRVANEAAEAFRKAEDGGVDEKESKGVVNEEKGCVEMEGSS
NLFYLDEEEIFEMPRLLDDMAEGLMLCPPQCLDGYMDWNDVGTDDDLKLWSFTI*
>PpCBF5
MNRFFSHFSDSVDQPESSSLSDATVTTLRASWSDEDVILASSRPKKRAGRRVFKETRHPVYR
GVRRRNNDKWVCEMREPKKTKSRIWLGTYPTAEMAARAHDVAALAFRGKLACLNFPDS
AWRLPVPASMDAMDIRRAAAEAAEGFRPVEFGGVSSSSSDEKERMVVQVEEKKKKKDSV
KMEKSRSLSLSYWDEEEVFDMPRLLDDMAQGLLLTPPQCLGGDIWDDMGTDADVKLWSF
SN
*
>PpDREB2C
MGAYDQGSNEVALTLDSTRKRKTRSRRDGTSVAETLEKWKEYNKQLESDNNEGKTRKVP
AKGSKKGCMKGKGGPDNSRCNYRGVRQRTWGKWVAEIREPNRGSRLWLGTFPTALDAA
LAYDEAAKAMYGPAARLNLPNVANYSSWKESSQETSSATTPSGSSAVATPGCSGSTSSSNH
SEVCADEDSKGFLDVKTENGEGESAIHAWSNAVNQALVKEETKDEFTEGYFHNDQPAVK
PEAYVGDFNWAGGQYTGDYLENFTEEEVFDVDELLSPLDDTPVRNPEPEQSLGSDVDAKL
LGSLNHMEQGPSGGEYNFDFLKRDDVTGEDDQGYYNLGLSDLGLGDFTGGTMQPEDGSY
NFSMDM*
>PdCBF
MVMDMIFAQVSDSADQPRSSSSSDASVTTLRTSDVILASSSPKKRAGRRVFKETRHPVYRGV
RRRDNNKWVCELRQPNKKKSGIWLGTYPTAEMAARAHDVAALAFKGKLACLNFADSG
WRLPVAASMDSMDIQRAAAEAAEGFRPVEFGGVSSGSSDEKERMVVVEEKKKKQAIVDM
GKSCSRLNLFYLDEEEMFDMPRLIDNMAEGLLLSPPQCLAGYLNWDDMETEADPKLWSFS
I*
>PfCBF
MVMDMIFAQVSDSADQPRSSSSSDASVSTLRTSDVILASSSPKKRAGRKVFKETRHPVYRGV
RRRDNNKWVCELRQPNKKKSGIWLGTYPTAEMAARAHDVAALAFKGKLACLNFADSG
WRLPVAASMDSMDIQRAAAEAAEGFRPVEFGGVSSGSSDEKERMVVVEEKKKKQAIVDM
GKSCSRLNLFYLDEEEMFDMPRLIDNMAEGLLLSPPQCLAGYLNWDDMETEADSKLWSFS
I*
>PaCBF
MDMFFSQLSDSVDQPQSSLLSDASVTTRGASCSDGDVILASSRPKKRAGRRVFKETRHPVYR

GVRRRNNDKWVCEMREPNKKKSRIWLGTYPTAEMAARAHDVAALAFRGKLACINFADS
AWRLPVPASMDTMDIRRAAAEAAEGFRPVEFGGVCSGSSDEKERMVVQVEEKNKKGSVN
LERSRSLSLSYWDEEEVFHMPRLLHDMAEGLLLSPSQCLGGYMNLDDMGTDADVKLWSFS
I
>MdCBF1
MDGCSNYYVDPPGESSSASDTSRPMFLTLSDKEVLLASTCPKKRAGRKKFRETRHPVFRGV
RRRNSGKWVCEVREPNKKTRIWLGTFPTAEMAARAHDVAAIALRGRSACLNFADSARRL
PVPASSNAKDIQTAATEAAEAFRPTEESDEVCGSPVAAPSESTSASEQQQVFYMDEEAVFG
MPGLLADMAEGMLLPPPHYSDDDDVDVCADVPLWSFSI
>MdCBF2
MNTIFSTQLSDSSEKPESSSDDTSITAQSQLASFSDEEVILASSRPKKRAGRRVFKETRHPVYR
GVRRRNNNKWVCEMREPNKKKSRIWLGTYPTAEMAARAHDVAALAFRGKLACLNFADS
AWRLPVPASIDSVDIRRAAAEAAETFRPAEFGGVLESGDDEXESKKMEEEKDCGGVEGSGI
LFYLDEEEMFDMPRLLDSMAEGLLLSPPHSSGGYMNWDDMGSNDDVSLWSFSN
>MdCBF4
MNTISRQLSDSAERPESSSESVTXRSQPTSFSDEEVILASSTPKKRAGRRVFNETRHPVYRGVR
RRNNDKWVCEMREPNKKKSRIWLGTYPTAEMAARAHDVAALAFRGRLACLNFADSAW
RLPVPASTDSVDIRRAAAEAAETFRPAEFGGVSESGDDEKESKKMEGEKDCGCAEQSDCG
GAEQSDCGGAEQSGSSFYLDEEEMFAMPRLLDSMAEGLLLSPPRRSAGSNMNWDDMGSN
DDDVNLWSFSK
>MdCBF5
MDAFSHYYVDPQPESSSASDANSSRPMFMNLSDEEVLLASTYPKKQAGRKKFKETRHPVY
RGVRRRNSGKWVSEVREPNKKTRIWLGTFPTAEMAARAHDVAAIALRGRSACLNFADSA
WRLPVPASANAKDIQTAAAEAAEQFRPVEESDEACGASAVAPSECTSAPEQQVHFMDEEA
VFGMPGLLANMAEGMLLPPPHYNADDVVDVYADVPLWSFSI
>EgCBF
MNSSSYISHPNSFSFDFAEPPFSLLLSHDRSAAPGNFSGEEVRLASDHPKKPAGRKKFRETRH
PVYRGVRLRDSGKWVCEVREPRKKSRIWLGTFPTADMAARAHDVAALALRGQSACLNFA
DSAWRLPVPASPNTKDIQKAAAKAAEAFQLLESEDVMSGNEKKLHSEEGVLYDEEDIFGM
PGLLANTAEGMLLSPPECSGDLYAGLDDGNLDAYASLWSYSM*
>HrCBF
MATLHLLCVIPHPASRKRHEPFRPKRDSSCPKKPAGRKKFRETRHPVYRGVRLRKSGKWVC
EVREPNKKSRIWLGTFLTAEIAARAHDVAAIALRGKSACLNFADSAWRLRIPETTCPKEIQK
AAAEAAVAFQAELNDTTADHGLDVEETIVEAIFTEESSEGFYMDEEFMFGMPTLWASMAE
GMLLPPPSVQFGPNYDFDGDADVSPLEY*
>CjCBF
MDSLSSWSSPESRVGNFSDEDVVLLASSNPKKRAGRKKFRETRHPVYRGVRRRDSGKWVC
EVREPNKKSRVWLGTFPTAEMAARAHDVAAIALRGRLACLNFADSSWRLPLPASTDPKDI
QKAAAEAAEAFRPEKDLRRGDDKMDEEEAAERTTMSDDVFFMDEEAVFGMPGLLTNMA
EGMLLPPPPPPQSNCGGYEEDDVESNADVSLWSYSV
>NtDREB1
MTSFSAFSELLGSEHESPVTLGEEYCPKLAAGCPKKPAGRKKFRETRHPVYRGVRLRNSGK
WVCEVREPNKKSRIWLGTFLTAEIAARAHDVAAIALRGKSACLNFADSAWRLRIPETTCPK
DIQKAAAEAAVAFQAEINDTTTDHDFDVEETIVEAIFTEENNDGFYMDEEESMFGMPSLVA

SMAEGMLLPPPSVRFEHNYDFDGDADASLWSY*
>NtDREB2
MDIFRSYYSDTLTESSSLSENSSSSRNRANLSDEEVMLASNNPKKRAGRKKFRETRHATYRG
VRKRNSDRWVCEIRQPNKKSRIWLGTYPTAEMAARAHDVAAIALRGRSACLNFADSVWK
LHIPASADAKDIQRAAAEAAEAFGRPSESEEADSGTTPETPESSKLFMDEEALFCMPGLLAN
MAEGLMLPPPHYVEVGDYVEAAADMPQWSYTM*
>NtDREB3
MEMCRSYYSDPLADSSSLSDSSSSCNRAIRSNEEVMLASNNPKKRAGRKKFRETRHPVYRG
VRKRNSGKWVSEVREPNKKSRIWLGTFPSAEMAARAHDVAAIALRGRSACLNFADSAWK
LPVPASSDAKNIQKAAAEAAEAFRSSEAENMPEYTGEDSKEVNTTPENMFYMDEEALFCM
PGLLANMAEGLMLPPPQCSQIGDDHMEADMPLWSYSI*
>NtDREB4
MNIFGDHNFDPLIPTLSTSLLPAAESSTSSDSGSSGSTPNYSDEEVMLASNYPKKRAGRKKFR
ETRHPVYRGIRRRNSNKWVCEVREPNKKSRIWLGTFPTAEMAARAHDVAAIALRGRSACL
NFADSAWRLPIPTSAAAKDIQKATVEAAETFRPVESHRENFKEIIVDQVIQELVAELPDNVL
FMDEEALFCMPRLLVNMAEGLMLPPPQCIIDGYEMEADHADMSLWSY*
>AtCBF1
MNSFSAFSEMFGSDYEPQGGDYCPTLATSCPKKPAGRKKFRETRHPIYRGVRQRNSGKWVS
EVREPNKKTRIWLGTFQTAEMAARAHDVAALALRGRSACLNFADSAWRLRIPESTCAKDI
QKAAAEAALAFQDETCDTTTTNHGLDMEETMVEAIYTPEQSEGAFYMDEETMFGMPTLL
DNMAEGMLLPPPSVQWNHNYDGEGDGDVSLWSY*
>AtCBF2
MNSFSAFSEMFGSDYESPVSSGGDYSPKLATSCPKKPAGRKKFRETRHPIYRGVRQRNSGK
WVCELREPNKKTRIWLGTFQTAEMAARAHDVAAIALRGRSACLNFADSAWRLRIPESTCA
KEIQKAAAEAALNFQDEMCHMTTDAHGLDMEETLVEAIYTPEQSQDAFYMDEEAMLGM
SSLLDNMAEGMLLPSPSVQWNYNFDVEGDDDVSLWSY*
>AtCBF3
MNSFSAFSEMFGSDYESSVSSGGDYIPTLASSCPKKPAGRKKFRETRHPIYRGVRRRNSGKW
VCEVREPNKKTRIWLGTFQTAEMAARAHDVAALALRGRSACLNFADSAWRLRIPESTCAK
DIQKAAAEAALAFQDEMCDATTDHGFDMEETLVEAIYTAEQSENAFYMHDEAMFEMPSL
LANMAEGMLLPLPSVQWNHNHEVDGDDDDVSLWSY*
>AtCBF4
MNPFYSTFPDSFLSISDHRSPVSDSSECSPKLASSCPKKRAGRKKFRETRHPIYRGVRQRNSG
KWVCEVREPNKKSRIWLGTFPTVEMAARAHDVAALALRGRSACLNFADSAWRLRIPETTC
PKEIQKAASEAAMAFQNETTTEGSKTAAEAEEAAGEGVREGERRAEEQNGGVFYMDDEA
LLGMPNFFENMAEGMLLPPPEVGWNHNDFDGVGDVSLWSFDE*
>AtCBF5
MENDDITVAEMKPKKRAGRRIFKETRHPIYRGVRRRDGDKWVCEVREPIHQRRVWLGTYP
TADMAARAHDVAVLALRGRSACLNFSDSAWRLPVPASTDPDTIRRTAAEAAEMFRPPEFS
TGITVLPSASEFDTSDEGVAGMMMRLAEEPLMSPPRSYIDMNTSVYVDEEMCYEDLSLWSY
*
>AtCBF6
MNNDDIILAEMRPKKRAGRRVFKETRHPVYRGIRRRNGDKWVCEVREPTHQRRIWLGTYP
TADMAARAHDVAVLALRGRSACLNFADSAWRLPVPESNDPDVIRRVAAEAAEMFRPVD

LESGITVLPCAGDDVDLGFGSGSGSGSGSEERNSSSYGFGDYEEVSTTMMRLAEGPLMSPPR
SYMEDMTPTNVYTEEEMCYEDMSLWSYRY*
>VrCBF1
MDSDHEEFSASSSSSSSRTNSNPSDSLLPLQCIGHKRKAGRKKFRETRHPIYRGVRQRNGNK
WVCEVREPLKKSRIWLGTFPTPEMAARAHDVAALALRGRFASLNFPDSAWRLXRPKSSSA
EDIQVAALEATKAFNPTAPSSSSLASALDNMSGVADSKKVLETSPNVESPKLKSQRMVLEV
SPVDTKRSEKVGDGSTPXFMDEEAMFNMQGLINSMAEGLLLTPPAMCKGFSWDDATDSHI
DLSLWNDD
>VrCBF2
MDLDRESSASSTSSPSRANPVSSDPRCPSRCIPHKXKTGRKKFRKTRHPIYRGVRQRNENKW
VSEVREPSKKSRIWLGTFPTPEMAARAHDVAALALRGHFASLNFPDSAWRLPRARSSSAGD
VQFAAIQAAKAFQQPPSSSSTPFVXDNMSAGSRKILETSSVVDTPQLKSQKKVVGVSSVDS
KSXEKAGDGFPTAFVDEEAVFNMPGLIDSMAEGLLLTPPAMCEGFSWDDAVSHIDLSLWN
HDFLS
>VrCBF3
MESERDQSSPSSSSSSSQTKCSISSSPVHKRKAGRKKFRETRHPVYRGVRQRNGNRWVCEMR
DPKNKSRIWLGTFPTPEMAARAHDVAALAFRGDFAALNFPDSTSRLPRAKSSSAGDIRVAA
LAAAMAFRPAAPSSSSSYISHVTACSEELETSCSEDSPQLESRKKVVGVALEDSESSEGSPHGS
STVFMDEEALFNMPGLINSMAEGLLLAPPTMLGGFSWDDTTSYTDLSLWNDD
>VrCBF4
MNTTSPPYSDPHPLVCNWDSLNLPDSDGGSEELMLASTHPKKRAGRKKFRETRHPVYRGV
RRRNSGKWVCEVREPNKTSRIWLGTFPTAEMAARAHDVAALALRGRGACLNFADSAWR
LHVPSSRDAKDIQKAAAEAAEAFRPMENDGVMQDERREESEVRTPDNVFVMDEEDVFGM
PGLLVNMAEGLLMPPPHSVADGYGGDDMAADADMSLWSYSI
>PtCBF1
MLASRNPKKRAGRKKFRETRHPVYRGVRRRNSGKWVCEVREPNKKSRIWLGTFPTAEMA
ARAHDVAALALRGRSACLNFADSAWRLPVPASSEAKDIQKAAAEAAGGFRPEGCVGGEL
MRTGDEGEKAAETTAEAGEEVFYMDDEAVFGMPGLLANMAEGMLLPPPHCGGGGDGW
DNMENIDADMPLWSFSI
>PtCBF2
MDVFCSYSDQNPIGSMSLLSVLDEQECSYSSVLSDSSITSSVTKGVQPGAIFSDEEVILASRNP
KKRAGRKKFRETRHPVYRGVRRRNSGKWVCEVREPNKKSRIWLGTFPTADMAARAHDV
AALALRGRSACLNFADSAWRLPTPASSDAKDIQKAAAEAAEAFRPEGSLGVELTRTGDEV
EKVAGTAAGDVFYMDDDADFGMPGLLANIAEGMLLPPPNCCGYSGGDSLDNMENNDT
DMSLWSFSV
>PtCBF3
MVMGGSNSFSPDKQESSLSSLLSDSSGSQQDSPSSNEKVLLATSRPKKRAGRRIFKETRHPIFR
GVRKRNGDKWVCELREPNKKSRIWLGTYPTPEMAARAHDVAALAFRGKSACLNFADSA
WRLPVPISNEAKDIRRAASEAAELFRTSDLGGQVMEDFRREDRGEVCSSTNDDIRDLPSENV
GYIDEEAEFNMPGLLASMAEGLLLSPPHYTGDWNDGEIDADWSLWSS
>PtCBF5
MEFENHSPSISSLRQNLSCKPCNNPINYIYNLKQAQERPVTVLKKKKAGRKKFKETRHPVY
RGVRKRNGNKWVCEVREPNKKSRIWLGTFTSPEMAARAHDVAALALKGETATLNFPDSA
LILPRAKSSSAGDIRRAARDAVEAFIPSASLCSPPSPSHHKNFSCVTSSSKVLSPALSDLNPHE

KGIENVFKPSLIGMQGVEVYGKKALNDDPFSMVMMSCPNNCSEKVSHQPNALFFDEEALF
NMPGLLDSMAEGLILTPPVMQRGFNWDDMACSTDLTLWEED
>PtCBF6
MCLLCPTYVTSCFMKNKAGRKKFKETRHPVYRGVRRRNGNKWVCEVREPNKKSRIWVGT
FKSPEMAARAHDVAALALKGELAALNFLDSALILPRAKSSSARDIQRAALAATEVLGRSAS
SCSSSSSPDHKKLSCVPNHSNATFFDEEALFNMPGLLDSMAEGLILTPPAMARGVYWDDM
ACSTDLTLWEDDYLDSQ
